# Ringed Seal (*Pusa hispida*) Haul‐Out Behavior and Emergence Timing in the Bering, Chukchi, and Beaufort Seas

**DOI:** 10.1002/ece3.72948

**Published:** 2026-01-25

**Authors:** Jessica M. Lindsay, Paul B. Conn, Peter L. Boveng, Justin A. Crawford, Lori T. Quakenbush, Andrew L. Von Duyke, Kristin L. Laidre

**Affiliations:** ^1^ School of Aquatic and Fishery Sciences University of Washington Seattle Washington USA; ^2^ Marine Mammal Laboratory Alaska Fisheries Science Center, NOAA‐NMFS Seattle Washington USA; ^3^ Arctic Marine Mammal Program, Alaska Department of Fish and Game Fairbanks Alaska USA; ^4^ North Slope Borough Department of Wildlife Management Utqiaġvik Alaska USA; ^5^ Polar Science Center, Applied Physics Laboratory University of Washington Seattle Washington USA

**Keywords:** climate change, denning, molting, phenology, satellite‐linked transmitters

## Abstract

Ringed seals are ice‐associated marine mammals that excavate lairs in snowdrifts on sea ice during winter for resting and pupping. Before lairs melt in the spring, seals shift from predominantly hauling out in lairs to predominantly hauling out on the surface of the ice to bask and molt, a transition we refer to as “emergence.” Emergence timing is poorly understood, which hinders planning and interpretation of aerial surveys for population monitoring. We used hidden Markov models (HMMs) to analyze haul‐out timelines from satellite‐linked transmitters to estimate emergence dates of ringed seals from 2005 to 2021 in the Bering, Chukchi, and Beaufort seas. Our HMMs were fit separately for adults and subadults and included a lair state and an emerged state, characterized by differences in diel behavior and the daily proportion of time seals spent hauled out. We explored covariates of the transition probability between states, including day of year, daylength, sea‐ice concentration, air temperature, and indices of snow melt progression. Emergence probability for adults was associated with longer daylengths and warmer temperatures. Median emergence date was 12 May (within 3 days of previous estimates from aerial surveys) and emergence date was positively associated with latitude. Subadults showed weaker seasonal changes in haul‐out behavior, potentially making HMMs more suitable for adults than subadults. Our results contribute to our understanding of ringed seal behavioral ecology, provide useful information for aerial surveys, and demonstrate the value of haul‐out data from tagged ringed seals for monitoring emergence timing in a warming Arctic.

## Graphical abstract

In the spring, ringed seals ( *Pusa hispida* ) shift from predominantly hauling out in lairs to predominantly hauling out on the surface of the sea ice to bask and molt, a transition we refer to as “emergence.” We used hidden Markov models (HMMs) to analyze haul‐out timelines from satellite‐linked transmitters to estimate emergence dates of ringed seals from 2005 to 2021 in the Bering, Chukchi, and Beaufort seas and to investigate the environmental variables associated with emergence probability. Emergence dates for adults were positively associated with latitude, and emergence probability was associated with longer daylengths and warmer air temperatures.

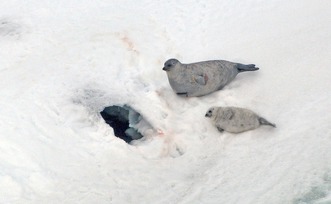

## 1 Introduction

**FIGURE 1:**
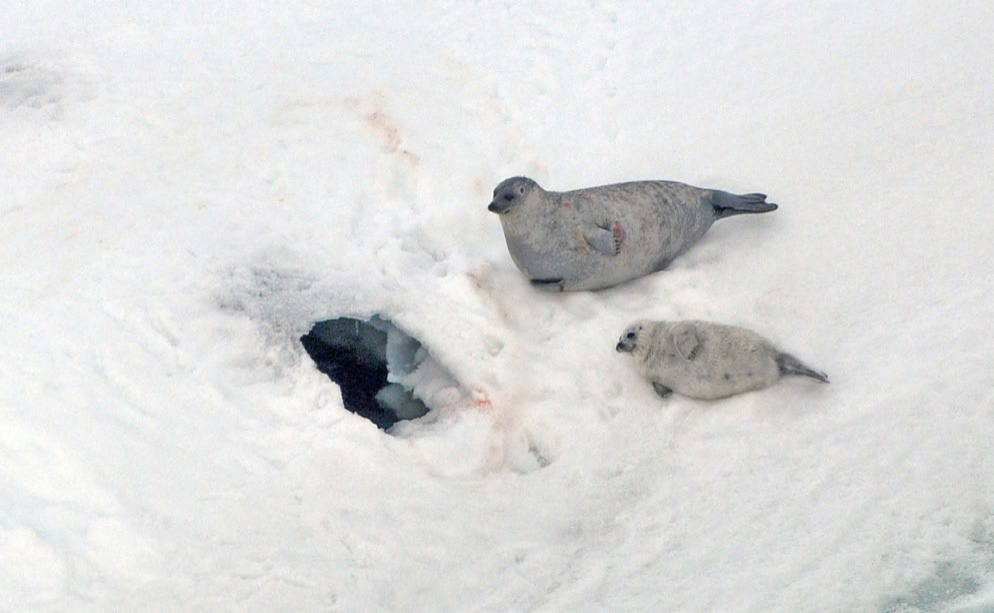
A ringed seal mother and pup hauled out next to a partially melted lair in the southeastern Chukchi Sea. Photo: J. Lindsay. NMFS MMPA Permit No. 19309. Reproduced from Lindsay et al. (2023).

In the Arctic, ice‐associated seals haul out on sea ice for resting, pupping, and molting (Kovacs and Lydersen 2008; London et al. 2024). “Hauling out” refers to temporarily exiting the water onto a solid substrate, and most ice‐associated seals haul out on the surface of sea ice. Ringed seals ( *Pusa hispida* ) are unusual, however, in that they spend part of their lives hauled out under the snow. During the winter, these seals excavate dens (“lairs”) in snowdrifts on the sea ice, providing a resting place that is insulated from extreme winter air temperatures (Smith and Stirling 1975). Ringed seals give birth to their pups in these lairs in March–April (Smith 1987), followed by a ~39‐day nursing period (Hammill et al. 1991). Foraging continues throughout the nursing period, and mating takes place under the ice before pups are weaned (Smith 1987; Kelly and Wartzok 1996). An insulated and dry place to haul out is especially important for young pups, who are born without blubber and are vulnerable to hypothermia. When wet, pups have a relatively warm lower critical temperature of 0°C (i.e., the temperature below which they must expend additional energy to maintain body heat) (Taugbøl 1982; Smith et al. 1991). Lairs also provide protection from polar bears ( *Ursus maritimus* ) (Hammill and Smith 1991; Furgal et al. 1996) and, when pups are small, avian predators and foxes (Smith 1976; Lydersen and Smith 1989). Later in the spring, ringed seals shift from predominantly hauling out in lairs to predominantly hauling out in the open on the surface of the sea ice for basking and molting, a transition we hereafter refer to as “emergence” (Smith and Hammill 1981; Kelly and Quakenbush 1990). As seals emerge, they typically bask at breathing holes or cracks near the lairs that they used during the winter (Figure 1), leaving their lairs intact until melting snow eventually causes the ceilings to collapse (Kelly and Quakenbush 1990; Kelly 2022; Quakenbush & Crawford pers. obs.). Seals may also bask at lair sites after excavating through the snow covering the lair (Smith and Hammill 1981; Kelly, Badajos, et al. 2010). Emergence timing can vary by individual, location, and year (Kelly et al. 2006), but information on the environmental factors associated with this transition is limited. Filling this knowledge gap is necessary for anticipating how future warming may impact ringed seals.

Ringed seals were listed as “threatened” under the U.S. Endangered Species Act in 2012 due to anticipated impacts of climate change, particularly the concern that declining snow depth and earlier melt could either preclude lair construction or force ringed seals to emerge from their lairs before pups can tolerate being exposed to thermal extremes or predators (NMFS 2012). In this context, monitoring for potential changes in ringed seal population size is a high priority for their conservation and management. Aerial surveys are a common method for monitoring ringed seal population size and for exploring how ringed seal densities vary with habitat variables (e.g., Frost et al. 2004; Bengtson et al. 2005; Ferguson et al. 2017; Boveng et al. 2025) but accurate estimates are hindered by availability bias, where an unknown fraction of the population is unavailable for detection (Marsh and Sinclair 1989; Pollock et al. 2006). In other diving marine species, data from satellite‐linked transmitters have been used to account for availability bias by estimating what fraction of the population is underwater and thus unavailable for detection (e.g., Heide‐Jørgensen and Laidre 2015; Nykänen et al. 2018; Heide‐Jørgensen and Lage 2022; Dunn et al. 2024). For ringed seals, lair use adds another layer of complexity. Only seals hauled out on the surface of the sea ice are available to be counted by the visual or infrared methods used in aerial surveys (Moreland et al. 2013; Boveng et al. 2025). Seals in the water—or out of the water, but concealed inside snow lairs—cannot be detected and counted (Boveng et al. 2025). Interpretation is further complicated by seasonal changes in haul‐out behavior that increase ringed seals' availability for detection. After emergence, not only are fewer seals concealed by snow cover, but seals also spend more time hauled out (Born et al. 2002; Kelly, Badajos, et al. 2010; Martinez‐Bakker et al. 2013; Crawford et al. 2018; Hamilton et al. 2018; Von Duyke et al. 2020). Additionally, they shift their diel behavior (i.e., the timing of haul‐out within a 24‐h period), spending more time hauled out around solar noon (Kelly and Quakenbush 1990; Kelly et al. 2006; Kelly, Badajos, et al. 2010; Von Duyke et al. 2020). These changes are thought to facilitate molting, which is an energy‐intensive process that requires elevated skin temperatures for tissue regeneration (Feltz and Fay 1966; Thometz et al. 2023). Water is 25 times more conductive than air, so spending more time hauled out significantly reduces heat loss for molting seals relative to staying in the water (Boily 1995; Thometz et al. 2023). Seals can further minimize heat loss by hauling out at midday when ambient temperatures and solar radiation are highest (Walcott et al. 2020). As spring progresses, the number of seals available to be counted increases, with some day‐to‐day variability due to weather. Previous studies have found that seal counts increase with survey date, air temperature, and melt progression and decrease with wind speed (Smith and Hammill 1981; Moulton et al. 2002; Frost et al. 2004; Kelly et al. 2006; Lindsay et al. 2021). For improved aerial survey planning and interpretation, and to better anticipate how emergence timing may be affected by climate change, further research is needed on the timing and drivers of emergence and the differences in haul‐out behavior before and after emergence.

To our knowledge, the only two studies to compare haul‐out behavior in lairs with haul‐out behavior after emergence were conducted in the Chukchi and Beaufort seas in 1983–1984 and 1999–2003 using a combination of VHF radio transmitters and round‐the‐clock visual monitoring to determine when seals were hauled out in lairs and when they emerged onto the surface to bask (Kelly and Quakenbush 1990; Kelly et al. 2006; Kelly, Badajos, et al. 2010). This approach provided valuable evidence that ringed seal emergence is associated with an increase in time spent hauled out and a shift in diel behavior. However, this approach was labor‐intensive and could only be used in the nearshore landfast ice where seals were within range of radio receivers and visual observers. In contrast, satellite‐linked transmitters (hereafter “tags”) with wet/dry sensors can provide information on seal movement, haul‐out, and dive behavior throughout their range, and sometimes for multiple years. Tagging studies have identified seasonal patterns in haul‐out behavior (e.g., Crawford et al. 2018; Hamilton et al. 2018; Von Duyke et al. 2020), but the wet/dry sensors in current satellite‐linked tagging technology do not identify whether a seal is hauled out on the sea ice or hauled out inside a snow‐covered lair. However, based on the behavioral shifts identified in the VHF‐ and visual‐based emergence studies described above (Kelly and Quakenbush 1990; Kelly et al. 2006; Kelly, Badajos, et al. 2010), we propose that lair usage may be estimable from the amount of time seals spend hauled out of the water each day and the time of day at which they do so. In turn, emergence timing may be estimable by identifying when ringed seals switch their behavior from one haul‐out pattern to another.

In this study, we analyze the seasonal changes in haul‐out behavior of satellite‐tagged ringed seals as a novel way to estimate the timing of emergence and to quantify ringed seal haul‐out behavior before and after emergence. We apply hidden Markov models (HMMs; Zucchini et al. 2016) to a ringed seal haul‐out dataset collated from previous studies in the Bering, Chukchi, and Beaufort seas during 2005–2021 to determine when seals switched their haul‐out behavior from hauling out in lairs (a “lair state”) to emerging on the sea ice (an “emerged state”), and to identify environmental variables associated with this transition. Our motivations were to better understand ringed seal behavioral ecology during this important period, to help inform aerial survey planning and interpretation, and to develop a method for monitoring emergence timing in a warming Arctic.

## 2 Materials and Methods

### 2.1 Ringed Seal Haul‐Out Data

**FIGURE 2:**
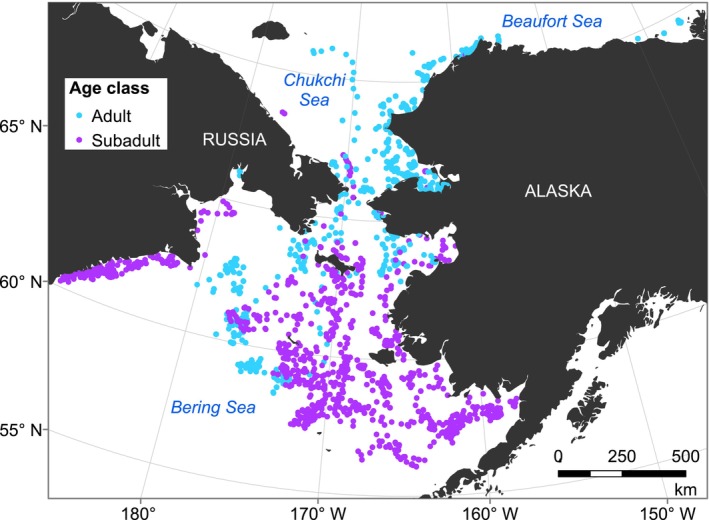
Daily locations of adult (*n* = 55) and subadult (*n* = 16) ringed seals instrumented with satellite‐linked transmitters. Seals were tagged during 2005–2019 and points represent daily locations between February–June 2005–2021.

We acquired ringed seal haul‐out data from previous tagging projects in the Bering, Chukchi, and Beaufort seas conducted by the National Oceanic and Atmospheric Administration (NOAA; Kelly, Badajos, et al. 2010), the Alaska Department of Fish and Game (ADF&G; Crawford et al. 2012; Quakenbush et al. 2019), and the North Slope Borough (NSB; Von Duyke et al. 2020, unpubl. data) (Table S1). In these projects, seals were captured off the coasts of Alaska and Canada in the Chukchi and Beaufort seas (Chukchi 2005–2010, 2014, 2017; western Beaufort 2011–2019; eastern Beaufort 2006), and some seals subsequently moved into the Bering Sea (Figure 2, Table S1). Seal capture and tagging methods are detailed in Kelly, Badajos, et al. (2010); Crawford et al. (2012), and Von Duyke et al. (2020). Briefly, ringed seals were captured with nets set into breathing holes in the landfast ice during March–May, adjacent to ice floes in June–July, or adjacent to land in September–October. Captured seals were fitted with tags manufactured by Wildlife Computers, specifically SPLASH, and Smart Position and Temperature (SPOT) tags. SPOT tags were permanently attached to a hind flipper, while SPLASH tags were glued to hair on the head or back and fell off during the annual molt the following spring. All tag types provided estimated locations and hourly data on the percent time the tag was dry. For the present study, we included all ringed seals instrumented with tags that transmitted data at any time from 1 February through 15 June (*n* = 64), which we expected to encompass haul‐out behavior characteristic of both lair usage and emergence.

Demographic and biometric data collected during seal captures differed among projects and years, but capture records generally included sex, mass, and the number of claw bands (“annuli”) present on the foreclaws as an estimate of minimum years of age (Table S2). We classified seals as adults based on two criteria: a body mass threshold (≥ 35 kg; Crawford et al. 2015, ADF&G unpubl. data) and sex‐specific claw band thresholds corresponding with estimated ages of maturity for the region (≥ 4 claw bands for females, ≥ 5 for males; McLaren 1958; Frost and Lowry 1981; Crawford et al. 2015; Von Duyke et al. 2020, ADF&G unpubl. data). When both mass and claw band data were available (*n* = 43 individuals), these methods were generally in agreement. In 6 cases where seals exceeded the sex‐specific claw band threshold but weighed < 35 kg, we used the claw‐based age classification. For individuals with incomplete capture records (i.e., only claw bands (*n* = 2) or mass (*n* = 18) were recorded), we used whichever age classification metric was available. Following this classification system, our dataset consisted of 48 adults (21 females, 27 males) and 16 subadults (5 females, 11 males). The majority of adult seals (73%) in the dataset were tagged with SPOT tags, while the majority of subadults (81%) were tagged with SPLASH tags. Within each age class, there was no apparent bias in tag type between sexes. Of tags deployed on these 64 individuals, most transmitted data during February–June of only one year, but tags deployed on 7 adults (1 female, 6 males) transmitted data during two successive years.

### 2.2 Modeling Emergence Probability

#### 2.2.1 Data Preparation and Environmental Covariates

Dry readings in the hourly percent dry timelines recorded by SPLASH or SPOT tags can be generated when a seal hauls out or, particularly for SPLASH tags placed on the head, when a seal briefly raises its tag above the water while surfacing between dives. To isolate haul‐out behavior from surfacing behavior, we applied criteria from Irani et al. (2024) to the hourly percent dry timelines of both tag types. Specifically, we considered hourly percent dry values to represent the hourly percent hauled out if a given hour was ≥ 50% dry, or < 50% dry but adjacent to an hour with ≥ 95% dry. For hours that did not meet these criteria, we set the hourly percent hauled out to 0%. This process converted the raw “hourly percent dry” data to “hourly percent hauled out” and ensured that our analysis would not be biased with respect to tag placement or age class. Tags placed on the head or back (which were more common for subadults in our dataset) would be more likely to record a “dry” reading when seals surface to breathe, whereas flipper tags generally stay submerged unless a seal completely hauls out of the water. We also restricted our analysis to daily haul‐out records containing a full 24 h of data to prevent sampling bias.

For each combination of individual seal and year (71 total, including 55 adults and 16 subadults), we then summarized hourly haul‐out records into two daily variables. First, we calculated “proportion of day hauled out” for each seal as the sum of the hourly percentages of time hauled out divided by 24. Second, to assess diel behavior, we calculated “peak haul‐out hour” as the weighted mean time of day at which the seal was hauled out. To do this, we converted each hour of the day into solar time, where the sun is highest in the sky at solar noon. We then converted solar hour into radians (range –π to π) to facilitate circular calculations, maintaining continuity between 00:00 and 24:00 and defining solar noon (12:00) as 0 rad. Next, we calculated the weighted circular mean of the seal's haul‐out bouts for that day, where each hour of the day was weighted by the amount of time that the seal was hauled out during that hour. We took this value to be the seal's peak haul‐out hour. If a seal spent 0% or 100% of a day hauled out, we set its peak haul‐out hour for the day to NA.

To associate haul‐out data with environmental variables, we estimated daily locations for each seal. We first applied a speed‐distance‐angle filter (Freitas et al. 2008) to the raw Argos locations to remove biologically implausible and extreme outliers (e.g., locations that would have required a seal to swim at impossible speeds between one location and the next), which can occur when tag transmissions are interrupted or limited during a satellite pass (CLS 2016). We then took a weighted average of the seal's remaining locations within each day, such that locations were inversely weighted by the Argos error radius (London et al. 2024). Data could only be transmitted when Argos satellites were present and when tags were out of the water, that is, when seals were hauled out or, for seals instrumented with SPLASH tags, at the surface of the water. To minimize data loss when these conditions were not met, data were stored on the transmitters for several days to provide opportunity for transmission before being overwritten by new data. Gaps of up to 2 weeks between transmissions were common for both tag types, and some SPOT tags had gaps of up to 2 months. Previous analyses of haul‐out data from tagged ice‐associated seals have shown that missing data do not substantially bias estimates of haul‐out behavior (Conn et al. 2012). In all cases where a tag resumed transmitting after a data gap, we were confident that the tag was still attached to a live seal due to variable haul‐out data (both peak haul‐out hour and daily proportion hauled out) that were consistent with other ringed seals in the dataset (Figures S2 and S3). Our discrete‐time modeling framework required that observations were evenly spaced in time (e.g., daily) (McClintock and Michelot 2018), so we filled in missing dates in each time series such that each seal had a contiguous daily record between its first and last transmission date within a given year. We did not attempt to interpolate or otherwise estimate haul‐out observations for these missing dates (i.e., some seals were left with many “NA” values for proportion of day hauled out). Missing daily location estimates for each seal were filled in with the same coordinates as the seal's previous daily location (London et al. 2024) because ringed seals occupy small home ranges prior to sea‐ice breakup (Kelly, Badajos, et al. 2010). If location estimates were missing at the start of an individual's time series, we instead used its first available location from that year. In one case where an adult seal with 2 years of haul‐out data did not have any location estimates during February–June 2006, we assigned it the mean of its locations from February–June 2005 because ringed seals exhibit breeding site fidelity, and we therefore expected the seal to return to its home range from the previous year (Kelly, Badajos, et al. 2010). Having a complete time series of location estimates for each seal (including during gaps between haul‐out transmissions) enabled us to extract information on the environmental conditions each seal would have experienced throughout its full time series. Ringed seal daily movements during winter and spring (median home range < 1 km^2^; Kelly, Badajos, et al. 2010) are typically small relative to the coarse 25–32 km resolution of environmental covariates, so we do not anticipate appreciable bias from this procedure.

We selected a set of environmental variables that we hypothesized would influence emergence timing and matched these with seal haul‐out records by date and location. We expected that decreasing sea‐ice concentration, warming air temperatures, and melting snow cover would make seals more likely to emerge (i.e., begin basking) due to the need for molting seals to maintain elevated skin temperatures, and lairs beginning to collapse (Kelly et al. 2006; Thometz et al. 2023). Daily sea‐ice concentration values were obtained from the National Snow & Ice Data Center SSM/I passive microwave data (https://nsidc.org/data/nsidc‐0051) at 25‐km resolution (Cavalieri et al. 1996, updated yearly). Daily mean air temperatures (°C at 2 m above sea level) were obtained from the North American Regional Reanalysis (NARR) dataset at 32‐km resolution (Mesinger et al. 2006). For each 32‐km cell within the air temperature dataset, we calculated cumulative thawing degree days as the number of days since 1 April with a mean daily temperature of ≥ 0°C. We also calculated a rolling 7‐day average of daily temperature within each cell. These two air temperature variables reflected the possibility that emergence could be related to broader temporal trends in air temperature rather than day‐to‐day variation. We also obtained 25‐km resolution melt onset dates from the NASA Cryosphere database (Markus et al. 2009; https://earth.gsfc.nasa.gov/cryo/data/arctic‐sea‐ice‐melt), including both early and continuous melt onset. Early melt onset represents the day at which the snow and sea‐ice first begin to thaw, with the potential for subsequent freeze/thaw cycles, while continuous melt onset represents the day at which the snow and sea‐ice are in a melt state for the remainder of the spring (Markus et al. 2009). We used spatial interpolation (ordinary kriging via the autokrige() function from the automap package; Hiemstra et al. 2009) in Program R (R Core Team 2017) to fill in missing melt values in the southern portion of our study area and along coastlines. We matched each seal haul‐out location with the melt onset date from the corresponding grid cell, and then subtracted the melt date from the haul‐out date as an index of melt progression. For example, if the melt index for a seal haul‐out record was −5 or + 5, this would indicate that the haul‐out data was recorded 5 days before melt or 5 days after melt, respectively.

#### 2.2.2 Hidden Markov Models

We analyzed the ringed seal haul‐out data using hidden Markov models (HMMs; Baum and Petrie 1966; Baum and Eagon 1967). Briefly, HMMs are discrete time series models composed of an unobserved (“hidden”) state sequence and an observation sequence, where the probability distributions of the observed variables are dependent on the underlying state (Auger‐Méthé et al. 2021; Langrock et al. 2012; Zucchini et al. 2016). The state sequence itself is a Markov chain, such that the state at time *t* is dependent on the state at the previous timestep with some probability of transitioning between states. Transition probability can be constant or vary in association with covariates. Given a set of observation sequences (e.g., time series for multiple individuals) and a specified number of hidden states set by the user, the HMM framework allows for the estimation of (1) parameters for the state‐dependent probability distributions of each observed variable, (2) transition probabilities between states, and if applicable, (3) the effect of any covariates on those transition probabilities or on the state‐dependent probability distributions (e.g., Patterson et al. 2009; Michelot et al. 2017; Klappstein et al. 2023). The true state sequence is unknown, but it can be estimated using state‐decoding algorithms such as the Viterbi algorithm (Viterbi 1967). The Viterbi algorithm takes into account the state‐dependent probability distributions and transition probabilities estimated by the HMM, and then iterates over the observation sequences to identify the most likely sequence of underlying states.

The framework above is well‐suited for investigating ringed seal haul‐out behavior, where we assume that a seal's haul‐out behavior is dependent on its underlying behavioral state. We can thus use observation sequences (i.e., haul‐out records) to estimate when seals were exhibiting haul‐out behavior consistent with predominantly hauling out in lairs versus predominantly hauling out on the exposed surface of the sea ice or snow, and the timing of this behavioral transition. Because HMMs can incorporate covariates on transition probabilities between behavioral states, they are also ideal for our objective of exploring what environmental variables are associated with emergence.

**FIGURE 3:**
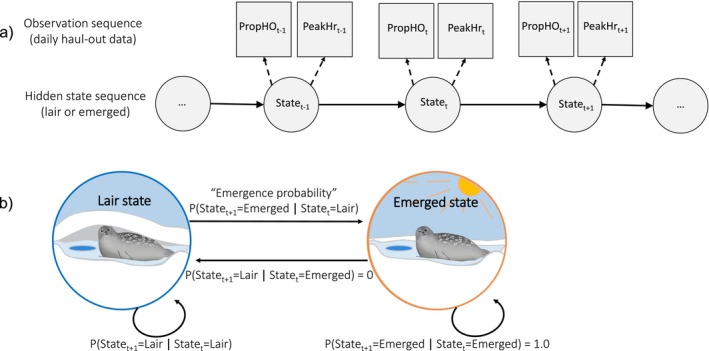
(a) Conceptual diagram of HMM state and observation sequences, modified from fig. 4d of McClintock et al. (2020). Squares in the top row represent a sequence of daily haul‐out observations from the satellite tags, including proportion hauled out (PropHO_t_) and peak haul‐out hour (PeakHr_t_). The observations at each daily timestep *t* are assumed to have been generated by a state‐dependent probability distribution corresponding to the seal's underlying hidden state (State_t_), either the “lair” or “emerged” state. (b) Diagram of state transitions and the daily probabilities of those transitions occurring. Once emerged, the probability of remaining in the emerged state is set to 1.0 and transitioning from the emerged state to the lair state is set to 0. The probabilities of remaining in the lair state and transitioning from the lair state to the emerged state (i.e., the daily “emergence probability”) are estimable.

We structured our HMMs to include two behavioral states: (1) the lair state, and (2) the emerged or basking state (Figure 3). All haul‐out records in February were labeled as belonging to the “lair” state, as seals are expected to be reliant on lairs during the coldest parts of winter. We also set the probability of staying in the emerged state to 1, such that seals could not switch from the emerged state back to the lair state. Kelly, Badajos, et al. (2010) observed that 60% of ringed seals returned to their lairs one or more times after first emerging onto the surface; however, these seals only spent 3% of their time in lairs after first emerging, suggesting that modeling emergence as a discrete process was a reasonable simplification. Observed variables included daily proportion hauled out and peak haul‐out hour as described above. Consistent with the HMM framework, both observed variables were assumed to have state‐dependent probability distributions, such that the estimated parameter values of the distributions differed between the lair state and emerged state. Based on previous studies, we expected that behavior in the lair state would consist of more time in the water (i.e., a lower proportion hauled out) and more time hauled out at night (i.e., a more nocturnal peak haul‐out hour) compared to the emerged state. Initial parameter values were based on reported estimates from Kelly et al. (2006) and then optimized via maximum likelihood (McClintock and Michelot 2018). Because the “proportion hauled out” data were bounded between 0 and 1 and contained exact zeros and ones (which cannot be modeled by a standard beta distribution), we used a zero–one‐inflated beta distribution for this observed variable. This allowed us to account for a probability mass at zero (i.e., when a seal spends none of the day hauled out) and a mass at one (i.e., when a seal spends all of the day hauled out). For the “peak haul‐out hour” observed variable, we used a von Mises distribution, which is a circular version of a normal distribution that allows for continuity between hour 0 (expressed in radians as –π) and hour 24 (π) (Zucchini et al. 2016). HMMs were constructed and fitted using the momentuHMM package (McClintock and Michelot 2018) in Program R (R Core Team 2017).

**FIGURE 4:**
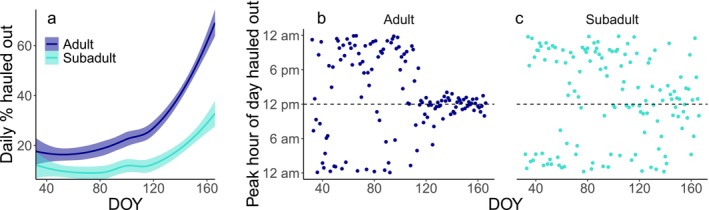
Temporal patterns in haul‐out behavior by day of year (DOY) for adult (*n* = 55) and subadult (*n* = 16) ringed seals during February–June. (a) Loess smooth and 95% confidence intervals for mean daily % hauled out taken across all individuals within each age class. Mean peak haul‐out hour taken across all individual adults (b) or subadults (c). Peak hour of day hauled out is scaled to solar time, such that 12 pm represents solar noon.

We produced separate HMMs for adult and subadult seals. Within each age class, observation sequences from all individual seals were used to estimate shared parameters for a single HMM. Ringed seals exhibit age‐related differences in habitat use, movement, diving behavior, and haul‐out behavior during fall–winter (Crawford et al. 2012, 2018), so we expected haul‐out behavior in spring could differ as well. Exploratory plots of seasonal patterns in the two observed variables supported behavioral differences between age classes (Figure 4). Within each age class, we calculated the daily means for proportion hauled out and peak haul‐out hour across all individuals during February–June. Subadults did not increase their time hauled out as much as adults and did not shift their peak haul‐out hour. Similar assessments did not identify substantial differences in seasonal haul‐out patterns between sexes in the overall dataset or between adult males and females (Figure S1). Accordingly, we did not produce separate HMMs for males and females of either age class.

For both age classes, we considered several candidate models to explore the effects of covariates on the daily probability of transitioning from the lair state to the emerged state, which we hereafter refer to as “emergence probability.” We included day of year (DOY) as a covariate because seals are more likely to emerge from lairs as the spring progresses. We considered a candidate model with an interaction between DOY and sex due to the potential differences in emergence timing observed in our exploratory plots and because molting timing differs between sexes in other pinnipeds due to pupping and lactation (Reder et al. 2003; Beltran et al. 2019). Of particular interest for aerial survey planning, we also included a candidate model with an interaction between DOY and latitude because seals at more northern latitudes may emerge later. We also considered a daylength variable (calculated from DOY and latitude for each haul‐out location) to evaluate whether emergence probability is associated with photoperiod.

The timing of ringed seal emergence within the same study area can vary interannually (Kelly et al. 2006), though the composite haul‐out dataset used in this study spans 16 years and thus could also include temporal trends related to climate change. To test for shifts in emergence timing, we included a candidate model with an interaction between DOY and year, where both variables were treated as continuous covariates. We also tested whether melt‐related covariates (i.e., melt indices, air temperature, cumulative thawing degree days) had a stronger effect on emergence than DOY. Conceptually, this is similar to an interaction between DOY and year, but with fewer parameters to estimate, because melt is occurring earlier in the Arctic (Bliss et al. 2017).

Due to our limited sample size and correlations between covariates, we generally considered only one environmental covariate on emergence probability at a time. However, we also tested models with both air temperature and daylength because both variables were associated with peak molt timing in Saimaa ringed seals (*Pusa saimensis*, formerly *Pusa hispida saimensis* ; Niemi et al. 2022). Our overall set of candidate models consisted of a null model (with no covariates) and models with DOY, DOY × sex, DOY × latitude, daylength, daylength × latitude, DOY × year, sea‐ice concentration, early melt index, continuous melt index, daily air temperature, daily air temperature + daylength, 7‐day rolling mean of air temperature, 7‐day rolling mean of air temperature + daylength, and cumulative thawing degree days as covariates (see model list in Table 1). In each candidate model, covariates had a logit‐linear effect on emergence probability (McClintock et al. 2020) and no effect on the state‐dependent probability distributions for daily proportion hauled out or peak haul‐out hour. We evaluated which model was the best supported for adults and subadults using Akaike's Information Criterion (traditional AIC; Akaike 1973), selecting the model with the lowest AIC as the best model (Burnham et al. 2011). Model validation was performed using quantile‐quantile plots of pseudo‐residuals for proportion hauled out and peak haul‐out hour (McClintock and Michelot 2018) and by a leave‐one‐out sensitivity analysis (see below in 2.2.3). We interpreted results using the models' estimates of the transition probability matrix and the state‐dependent probability distributions of daily proportion hauled out and peak haul‐out hour, and by visually assessing plots of the relationships between covariates and emergence probability (McClintock and Michelot 2018).

**TABLE 1:** ΔAIC values for candidate HMMs used to assess lair and emerged states based on haul‐out behavior for adult and subadult ringed seals. The best model for each group is indicated with an asterisk (*).

Covariates on emergence probability	Total No. of parameters	Adult ΔAIC	Subadult ΔAIC
Daily air temperature + daylength	16	0*	1.26
Daylength	15	2.26	0*
7‐day rolling mean air temperature + daylength	16	3.64	2.00
Daylength × latitude	17	6.17	2.50
DOY × latitude	17	14.01	3.35
7‐day rolling mean air temperature	15	15.41	11.88
DOY	15	15.46	8.66
DOY × sex	17	15.93	11.81
Daily air temperature	15	18.71	15.54
DOY × year	17	19.46	12.66
Continuous melt index	15	34.14	10.11
Early melt index	15	36.54	7.53
Sea‐ice concentration	15	63.86	20.04
None	14	65.57	18.30
Cumulative thawing degree days	15	67.51	15.54

*Note:* we used standard AIC rather than AICc (corrected AIC) for model selection because determining the effective sample size for autocorrelated time series data is complex, and the large number of observations in our haul‐out dataset suggested that AIC and AICc would provide similar results.

#### 2.2.3 Emergence Dates

The most likely sequence of behavioral states for each individual seal was estimated by applying the Viterbi state‐decoding algorithm to our fitted HMMs. As described above, the Viterbi algorithm uses the state‐dependent probability distributions and transition probabilities from the fitted HMM to predict whether each haul‐out observation was more likely to have been generated from the lair state or emerged state (Viterbi 1967; McClintock and Michelot 2018). If a seal's Viterbi‐decoded state sequence identified a transition from the lair state to the emerged state, we considered the date of this transition to be that seal's emergence date. Because the zero–one inflated beta distribution that we used for daily proportion hauled out does not have an easily interpretable “mean” parameter, we also calculated the mean daily proportion hauled out for all records assigned to the lair state and the emerged state to facilitate comparison with estimates from Kelly et al. (2006).

Within each age class, the Viterbi‐estimated emergence dates for individual seals were dependent on the underlying HMM parameters, which were estimated from the pooled haul‐out dataset of all adults or subadults. Because the Viterbi algorithm identifies the single most likely state sequence without explicit confidence intervals, we applied a leave‐one‐out sensitivity analysis to evaluate the robustness of the estimated emergence dates to the inclusion of specific individuals. To do this, we re‐fit the best HMM for each age class with each seal sequentially removed from the dataset and re‐calculated the emergence dates for the remaining seals. We then plotted the estimated emergence dates for each seal across all iterations of this process to evaluate whether its emergence date was consistent or if it varied depending on the other seals included in the model.

We were interested in further exploring spatiotemporal trends in emergence timing by latitude and year to inform aerial survey planning, so in addition to testing HMMs with latitude and year as covariates in our candidate model set as described above, we also performed post hoc regressions on the Viterbi‐estimated emergence dates from the best HMM for adult seals. For each seal, we extracted the seal's latitude for the date on which the seal was estimated to have emerged and then tested linear models relating emergence dates to latitude or year, and used corrected AIC (AICc) to determine the most parsimonious model (Burnham and Anderson 2002). Latitude and year were highly correlated (Spearman's correlation coefficient = −0.72, *p*‐value < 0.0001), as our dataset generally consisted of more seals at higher latitude earlier in the study period and more seals at lower latitude later in the study period, so we did not test a model with both latitude and year together. We then used our best linear model to predict emergence dates for ringed seals near Prudhoe Bay at 70.49°N, where emergence dates were previously recorded by Kelly et al. (2006) in 1999–2003.

## 3 Results

### 3.1 Adults

**FIGURE 5:**
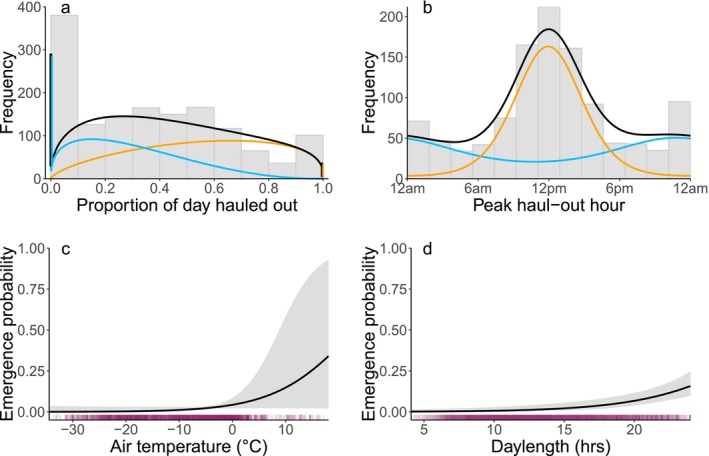
Summary of haul‐out behavior and emergence probability for adult ringed seals based on the best HMM. (a, b) Gray bars indicate the distribution of all observed haul‐out records for daily proportion hauled out and peak haul‐out hour, respectively. Hours are in solar time. Curves show the HMM‐estimated distribution for the lair state (blue), emerged state (orange), and all data (i.e., the two states combined, black). (c) Effect of daily air temperature on emergence probability (i.e., the daily probability of transitioning to the emerged state) when daylength is set to 18 h. (d) Effect of daylength on emergence probability when daily air temperature is set to 0°C. Shading indicates 95% confidence intervals. Purple tick marks along the *x*‐axes illustrate the distribution of observed values used in the model.

The best‐supported HMM for adult ringed seals included daily air temperature and daylength as covariates on emergence probability (ΔAIC ≥ 2.26 for all other candidate models; Table 1; Tables S3 and S4). Air temperature and daylength had positive effects on daily emergence probability. Emergence probability was ~0% when air temperatures were less than −10°C and when daylengths were less than ~10 h (Figure 5). Probability then increased rapidly above temperatures of ~0°C and daylengths of ~15 h. Daylength and temperature had a moderate variance inflation factor (VIF) of 2.12, and the direction and shape of the covariate effects shown in Figure 5 were consistent when compared to models with air temperature alone or daylength alone, suggesting that multicollinearity did not greatly impact our analysis.

Estimated distributions of haul‐out behavior for the lair and emerged states are shown in Figure 5 and Table S3. The HMM estimated that seals in the lair state spent a smaller proportion of their time hauled out of the water (mean = 18.8%, SD = 22.0%), sometimes remaining in the water for the entire day. In contrast, seals in the emerged state spent a substantially higher proportion of time hauled out (mean = 55.1%, SD = 25.2%), with some instances of individuals spending up to 100% of the day hauled out. For individual adults, the shift to spending more of the day hauled out tended to occur over the span of several days, and for many individuals, the proportion of time hauled out continued to increase after transitioning to the emerged state (Figure S2). The mean peak haul‐out hour was nocturnal for the lair state (10:48 pm solar time) and diurnal for the emerged state (11:48 am solar time). The time of day at which seals hauled out was more variable in the lair state, characterized by a diffuse von Mises distribution (i.e., low concentration parameter) compared to the highly concentrated emerged state (Table S2), and even within individuals, peak haul‐out hour varied from one day to the next (Figure S2). Temporal shifts in peak haul‐out hour were abrupt for some individuals and more gradual for others, occurring over the span of several days (Figure S2).

The Viterbi algorithm estimated emergence dates for 30 adult ringed seals, with a median emergence date of 12 May (mean = 6 May, SD = 21.8 days). Most seals without estimated emergence dates only had records from the earliest or latest part of the February–June period. There were 4 seals (3 females, 1 male; 2 SPLASH tags, 2 SPOT tags) with data of adequate seasonal coverage that were identified as being in the lair state for the full February–June period. These 4 seals did not show the strong seasonal increase in daily proportion hauled out that was observed in other adults, and/or they had one or more days in May–June where they spent 0% of the day hauled out. For the 30 adults with estimated emergence dates, mean air temperature at the time of emergence was −2.1°C (SD = 3.0°C). Mean daylength at time of emergence was 20.2 h (SD = 4.2 h), but seals at higher latitudes tended to emerge at longer daylengths compared to seals at lower latitudes (Spearman's correlation coefficient 0.76, *p*‐value < 0.001). For example, seals near Kotzebue Sound (latitude = 66.5°) emerged when daylength was ~16.9 h, and seals near Prudhoe Bay (latitude = 70.5°) emerged when daylength was ~22.5 h. Leave‐one‐out sensitivity analysis indicated that estimated emergence dates for individual seals were robust to removing one seal at a time from the dataset used to fit the HMM. Twenty‐seven adults each had completely consistent emergence dates across all possible leave‐one‐out iterations (i.e., their emergence dates did not change at all, or SD = 0; Figure S4). The seals that did demonstrate some sensitivity were still moderately consistent across leave‐one‐out iterations, with a maximum SD of 3.99 days for one individual and SD ≤ 1.2 days for all other adults.

**FIGURE 6:**
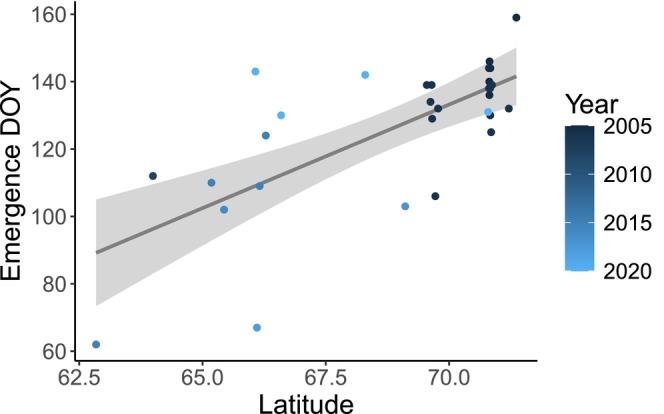
Relationship between latitude and Viterbi‐estimated emergence dates for adult ringed seals based on the best HMM. Blue points show emergence dates for individual seals (*n* = 30), with the shade of blue indicating the year that the seal's haul‐out data was collected. The linear regression line and 95% confidence interval for the mean relationship are shown in gray.

For the post hoc assessment of trends in emergence date, differences in emergence timing were best explained by latitude rather than year (ΔAICc = 13.29). Emergence occurred an estimated 6.2 days later for every degree of latitude (SE = 1.19, *p*‐value = < 0.001, Figure 6). There was no apparent correlation between the residuals from this regression and year (Spearman's correlation coefficient = 0.026, *p*‐value = 0.893). Our regression of emergence date by latitude predicted that seals in the Kelly et al. (2006) study area would have a mean emergence date of 16 May (95% CI of 9 May—23 May) (see Discussion for comparison with historical observations).

### 3.2 Subadults

**FIGURE 7:**
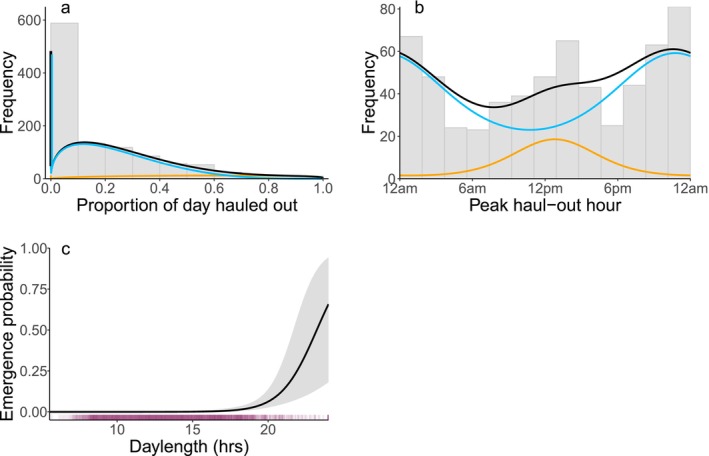
Summary of haul‐out behavior and emergence probability for subadult ringed seals based on the best HMM. (a, b) Gray bars indicate the distribution of all observed haul‐out records for daily proportion hauled out and peak haul‐out hour, respectively. Hours are in solar time. Curves show the HMM‐estimated distribution for the lair state (blue), emerged state (orange), and all data (i.e., the two states combined, black). (c) Effect of daylength on emergence probability (i.e., the daily probability of transitioning to the emerged state). Shading indicates the 95% confidence interval. Purple tick marks along the *x*‐axis illustrate the distribution of observed daylength values used in the model.

For subadults, the best‐supported HMM included daylength as a covariate on emergence probability (Table 1; Tables S3 and S4). Daylength had a positive effect on emergence, with an emergence probability of zero until daylength exceeded ~18 h (Figure 7). The HMM characterized the lair state as having a high probability that seals would spend 0% of their day hauled out, in contrast with a higher percent time hauled out in the emerged state (Figure 7); specifically, subadults spent an average of 13.0% (SD = 17.2%) of their day hauled out when they were estimated to be in the lair state, compared to 49.0% (SD = 29.8%) when in the emerged state. However, the HMM assigned most (91%) subadult haul‐out records to the lair state, and few (9%) records to the emerged state. The lair and emerged state both showed a diffuse distribution in the time of day at which seals hauled out, with weak peaks at 10:45 pm and 12:45 pm for the lair and emerged states, respectively. The Viterbi algorithm estimated emergence dates for 6 of 16 subadults, with a median emergence date of 27 May (mean = 28 May, SD = 7.9 days). Mean daylength at date of emergence was 19.1 h (SD = 1.3 h). At least 4 other subadults (2 females, 2 males; 4 SPLASH tags) had full‐season coverage of haul‐out data yet the model did not identify a transition to the emerged state. For the 6 subadults that did have estimated emergence dates, however, leave‐one‐out analysis indicated that their emergence dates were relatively insensitive to the other subadults included in the model (Figure S5). Three subadults had consistent emergence dates across leave‐one‐out iterations (i.e., SD = 0), while the remaining 3 exhibited only minor variation (SD range: 0.35–1.90 days). We did not perform post hoc regressions for subadult emergence dates due to the low number of individuals for which emergence date was identified.

## 4 Discussion

We applied HMMs to haul‐out data from tagged ringed seals to estimate the timing of the transition from using snow lairs to basking on the ice (i.e., emergence) and to quantify differences in haul‐out behavior before and after emergence. For adults, the patterns in haul‐out behavior estimated by our HMMs for the lair and emerged states are consistent with previous studies. Using a combination of radio tracking and visual observations, Kelly et al. (2006) determined that adult ringed seals in the Beaufort Sea hauled out for an average of 16% of the day while using lairs and 55% after emergence, corroborating our estimates of 19% (SD = 22%) and 55% (SD = 25%), respectively. The time of day at which seals hauled out was highly variable in the lair state (Figures 5 and 7), consistent with previous research (Kelly, Badajos, et al. 2010; Hamilton et al. 2018). Even within individuals, the time of day at which haul‐out occurred varied substantially from one day to the next (Figure S2). Despite this variability, as in previous studies, we found that seals in the lair state tended to haul out at night (Kelly and Quakenbush 1990; Kelly, Badajos, et al. 2010; Crawford et al. 2018; Von Duyke et al. 2020; Niemi et al. 2023). In the emerged state, adult ringed seals exhibited a more consistent diel pattern, with haul‐outs concentrated around solar noon (Kelly, Badajos, et al. 2010; Crawford et al. 2018; Von Duyke et al. 2020; Niemi et al. 2023).

Our estimated emergence dates for adult seals were highly variable (mean = 6 May, SD = 22 days), likely reflecting the broad latitudinal range of our dataset. The median estimated emergence date was 12 May, which aligns with a previous estimate of 15 May that was based on trends in ringed seal counts from aerial surveys in the Bering and Chukchi seas (Lindsay et al. 2021). Similarly, our analysis of emergence date by latitude (Figure 6) predicted that seals in the Beaufort Sea study area of Kelly et al. (2006) would have a mean emergence date of 16 May (95% confidence interval 9 May–23 May), which is within 5 days of the mean emergence date they observed in 1999–2003 (21 May, SD = 10.4 days; Kelly et al. 2006). Overall, the corroboration from previous studies suggests that our HMM for adults accurately represents haul‐out behavior and bolsters our confidence that the changes in haul‐out behavior detected by the HMM identify emergence from lairs.

Our models identified daily air temperature and daylength as the best predictors of emergence probability for adult ringed seals. Emergence probability was highest when daylength (i.e., photoperiod) was long and temperatures were warm. The changes in haul‐out behavior associated with emergence are thought to facilitate molting (Feltz and Fay 1966; Thometz et al. 2023) rather than reproductive behaviors, which occur earlier in the spring while seals are still using lairs (Smith 1987; Kelly and Wartzok 1996). Similar to our findings, the peak of molt in Saimaa ringed seals is associated with photoperiod and air temperature (Niemi et al. 2022). Longer photoperiod is known to induce molting in phocids and other taxa (Mo et al. 2000). The particular daylength or “critical photoperiod” at which organisms initiate processes like molting or reproduction is thought to be genetically inherited, and then environmental conditions like food availability and climatic variables influence the rate at which these processes proceed (Bradshaw and Holzapfel 2007). This is consistent with our model results, which suggest that both long days and warm temperatures are necessary for ringed seals to transition from using their lairs to basking on the surface of the sea ice.

Emergence probability increased as temperatures warmed, consistent with previous ringed seal studies in Alaska. Seals had a near‐zero probability of emerging when air temperatures were below −10°C (Figure 5), and the average temperature at which seals were estimated to have emerged was −2.1°C (SD = 3.0°C). Few seals were seen basking before temperatures exceeded 0°C in the Beaufort Sea in 1983 and −5°C in the Chukchi Sea in 1984 (Kelly and Quakenbush 1990), suggesting that the relationship between emergence and air temperature estimated by our model is reasonable. Because seals rely on elevated skin temperatures to facilitate molting, warmer air temperatures likely encourage seals to transition from using their lairs and begin basking at nearby breathing holes even when lairs are still intact (Kelly and Quakenbush 1990; Quakenbush et al. 2022). Warming air temperatures may also contribute to emergence by melting the snowpack and causing lairs to lose their structural integrity. For example, when snow depth was unusually low in Kotzebue Sound in 2019, ringed seals were seen hauling out in and next to lairs with ceilings that had melted open (Lindsay et al. 2023). In the Beaufort Sea in 1999–2003, emergence dates for tagged adult ringed seals exhibited a strong correlation with satellite‐derived melt onset dates (*R* ^2^ of 0.982, though based on only 5 data points) and the date at which the temperature at the snow‐ice interface reached 0°C (*R* ^2^ of 0.663; Kelly et al. 2006). The melt onset dataset used by Kelly et al. (2006) was unavailable for the full duration of our study period, and in situ measurements of snow temperature were also unavailable. Warming temperatures facilitate molting and degrade the snowpack simultaneously, so we cannot determine which of these mechanisms is more influential on emergence. Nonetheless, the associations among emergence date, melt onset date, snow temperature (Kelly et al. 2006), and now air temperature in our study suggest the potential for emergence to occur earlier in warmer regions or years.

Consistent with geographic patterns in air temperature, we found a positive relationship between adult emergence dates and latitude, with emergence occurring later in the spring for seals that were located farther north (Figure 6). While emergence was more closely associated with latitude than year in our dataset, we do know that air temperatures in the Arctic are increasing and snow and sea‐ice melt are occurring earlier in the spring. Across the Arctic as a whole, the date at which mean air temperature exceeded 0°C occurred 3.5 days earlier per decade during 2003–2017 (Bliss and Anderson 2018). Satellite‐based estimates of snow and sea‐ice melt onset are trending earlier as well (Markus et al. 2009; Bliss et al. 2019), and lairs are likely melting earlier as a result. However, warming trends vary among regions, and interannual variability can be substantial (Bliss and Anderson 2018; Bliss et al. 2019).

In the long term, warmer springs are expected to cause lairs to collapse before pups can tolerate being exposed on the ice, with negative effects on pup survival due to predation and hypothermia (Smith 1976; Lydersen and Smith 1989; Stirling and Smith 2004; Kelly, Bengtson, et al. 2010). To our knowledge, there have been no studies on whether ringed seals are shifting (or have the potential to shift) their birth phenology in response to changing conditions, as has been documented in other seal species (Cordes and Thompson 2013). To some extent, milder air temperatures may decrease the need for ringed seals and their pups to rely on lairs for thermal protection. However, young pups have a lower critical temperature of 0°C when wet, and premature lair collapse followed by freeze–thaw cycles could still be detrimental (Taugbøl 1982; Kelly et al. 1986). Concurrent with warming air temperatures, however, the proportion of pups in the ringed seal subsistence harvest by Alaska Natives in the Bering and Chukchi seas was higher during 2000–2023 than during 1960–1984, suggesting that warming has not had negative population‐level effects on pup survival in the region (Crawford et al. 2015; Quakenbush et al. 2024). It is unclear at what point earlier melt may begin to have negative population‐level effects on pup survival, or what melt onset timing is optimal for ringed seals.

### 4.1 Differences Between Age Classes

Subadult haul‐out behavior differed from adults, including a smaller seasonal increase in time hauled out and no seasonal shift in diel pattern, with most subadults appearing to stay arrhythmic throughout February–June (Figure 4). Discrete state models like HMMs may have been poorly suited for the subadult haul‐out data because they perform best when the differences between states are prominent. The decoded states for our subadult HMM only indicated emergence dates for the 6 out of 16 subadults with the most prominent seasonal patterns in haul‐out behavior. After emergence, these individuals spent a higher percentage of their time hauled out and focused their haul‐out bouts more closely around solar noon, similar to the adult seals (Figure S3). It is possible that these individuals were closer to adulthood, but their mean weight (25.9 kg) was not significantly heavier than that of the other subadults in our sample (23.0 kg; Student's *t*‐test *t*_13_ = 1.389, *p* = 0.094). The subadult HMM did not identify emergence dates for one‐half of the subadults that had haul‐out records throughout the full February–June period; these seals exhibited more subtle changes in behavior, or no change at all (Figure S3). Whereas most adult ringed seals were located in stable landfast ice, most subadults were located in seasonal pack ice at the southern margin of the study area in the Bering Sea (Figure 2), and at such low latitudes we would not expect any lairs to persist past mid‐June, so it is unlikely that our study period ended too early to include emergence. Rather, we suggest that a substantial proportion of subadults did not exhibit a strong enough seasonal change in haul‐out behavior for the transition to an emerged state to be detected by the HMM.

The weaker seasonal change in haul‐out behavior by subadults could be partly due to sample size (*n* = 16 subadults vs. *n* = 55 adults), but other explanations could include differences in lair usage, foraging, and physiology. Previous studies of ringed seals in the Bering, Chukchi, and Beaufort seas, using data that compose a substantial portion of our dataset, have found evidence of demographic habitat partitioning between ringed seal age classes, with adults overwintering at higher latitudes in areas with landfast ice and subadults overwintering at lower latitudes in areas with pack ice of lower concentration (Crawford et al. 2012; Von Duyke et al. 2020; but see Kelly 2022). These habitat associations suggest adult ringed seals select stable habitat that optimizes pupping and breeding at the expense of foraging opportunities, while subadults do not have reproductive constraints and are able to overwinter in less stable but more productive habitat (Grebmeier et al. 2006), potentially without the effort of constructing and maintaining breathing holes and lairs (Crawford et al. 2012; Ferguson et al. 2020). The thermoregulatory benefits of insulated lairs may be greatest for adult females that are rearing pups.

If subadults at the southern edge of the pack ice do not use snow lairs, or are less reliant on them compared to adults, it is possible that the seasonal changes in subadult haul‐out behavior that we observed represent a transition to basking (i.e., molting) without emergence from snow lairs. To our knowledge, no studies have investigated potential differences in lair usage between subadult and adult ringed seals, nor has it been possible to detect lairs in the Bering Sea pack ice where many subadults appear to overwinter (Figure 2). In the pack ice of the subarctic Sea of Okhotsk (south of our study area), at least some Okhotsk ringed seals ( *Pusa hispida ochotensis* ) shelter in the lee of ice hummocks rather than in lairs (Fedoseev 1965; Frost and Lowry 1981). If there are age‐ or habitat‐related differences in lair usage, this may explain why air temperature (and its probable influence on the snowpack) was less important for subadults. Additionally, if subadults already spend some or all of their time exposed on the sea ice without undergoing the same transition as adults, this could contribute to their more subtle seasonal change in haul‐out behavior.

Regardless of whether subadults at the southern edge of the pack ice use lairs, consistent access to prey throughout the winter may contribute to subadults having a weaker seasonal pattern in their haul‐out behavior. Even when adults and subadults were in the same location, Crawford et al. (2018) found that subadults made more frequent dives, hypothesizing that subadults may be less efficient foragers. Subadults may need to spend more time foraging (and thus less time hauling out) throughout the day to meet their energetic demands and to continue allocating energy to somatic growth. Alternatively, better access to prey may enable subadults to allocate more energy toward elevating their skin temperatures for tissue regeneration, rather than shifting their diel haul‐out behavior as strongly as adults to warm their skin in the midday sun.

Ringed seals of all age classes show seasonal fluctuations in body condition, with poorest body condition during and after molt (Quakenbush et al. 2020; Rosen et al. 2021). However, ringed seal subadults show less extreme seasonal fluctuations in body condition than adults (Ryg et al. 1990; Ferguson et al. 2020). This may be related to the differences in habitat and foraging opportunity described above, but even in captivity with consistent access to food, ringed seals ≥ 4 years old began exhibiting stronger seasonal patterns in food intake and body mass (Rosen et al. 2021). This suggests that differences in seasonality between age classes may have a hormonal physiological component which could, in turn, influence patterns in haul‐out behavior.

### 4.2 Uncertainty & Directions for Future Research

To apply HMMs to our ringed seal haul‐out data, we considered the lair and emerged states to be discrete, when in reality animal behavior is more complex. On days when weather is unseasonably warm or sunny, ringed seals may occasionally haul out on the surface of the sea ice instead of inside their lairs, and conversely, seals that have emerged and begun basking may reenter their lair if conditions become cold and windy again and the lair is still intact (Kelly, Badajos, et al. 2010). Within our dataset, some individuals displayed a gradual shift in haul‐out behavior over the course of multiple days (Figures S2 and S3), possibly reflecting intermediate levels of lair usage. We considered structuring our HMMs to allow seals to transition from the emerged state back to the lair state, or to have a third “intermediate” behavioral state. Ultimately, however, we chose to model emergence as a discrete, one‐directional process with only two behavioral states (functionally treating the HMM as a probabilistic change‐point model). This structural constraint (a) allowed us to compare our results with the in situ emergence study by Kelly, Badajos, et al. (2010), increasing our confidence in model performance, (b) provided more easily interpretable and biologically plausible covariate effects than HMMs with more complex structures, and (c) allowed us to more easily assess latitudinal and interannual trends in emergence dates.

Our approach assumes that seasonal changes in haul‐out behavior coincide with the transition from predominantly hauling out in snow lairs to more frequent basking behavior. This assumption is supported by previous studies in the region (Kelly and Quakenbush 1990; Kelly et al. 2006; Kelly, Badajos, et al. 2010) but may also be a simplification: it is possible that some changes in haul‐out behavior could have occurred before or after emergence, with potential decoupling between lair use and the onset of basking and molting. For example, Kelly and Quakenbush (1990) found that some ringed seals began to shift to diurnal haul‐out behavior while still using lairs. For subadults, it is possible that our HMMs detected molting‐associated changes in haul‐out behavior without lair usage. Future studies could supplement satellite tagging with aerial or on‐ice observations to confirm that changes in lair usage and changes in haul‐out behavior co‐occur, and to validate estimated emergence dates. Collaborations with Arctic Indigenous hunters who regularly observe basking seals while hunting or traveling on the sea ice should be considered (Gryba et al. 2021, 2025; Hauser et al. 2023) and additional variables such as cloud cover could be explored. Studies could also deploy tags with light and temperature sensors, which could provide information on when seals are hauled out in darker, insulated lairs versus exposed to sunlight and ambient air temperatures (Glass et al. 2021). This would provide additional evidence for how long into the spring ringed seals use lairs, and help determine whether lair usage differs between age classes. Additionally, cases of suspected lair usage identified from ringed seal tagging data could be joined with environmental data such as snow depth and ice topography to better quantify lair habitat requirements.

Comparisons of adult and subadult seasonal patterns in our study are partly confounded by differences in habitat, because most subadults were located at lower latitudes, which may have different sea‐ice concentrations, snow depths, and levels of primary productivity (Figure 2). The subadults in our sample were typically captured at lower latitudes (mean 68.28°N, median 66.99°N) than adults (mean 69.68°N, median 70.83°N) and then overwintered at lower latitudes as well. This latitudinal difference between age classes could be due to demographic habitat partitioning (Crawford et al. 2012; Von Duyke et al. 2020); but Kelly (2022) describes subadults as being common in the landfast ice in the Chukchi and Beaufort seas, so it is possible that this is somehow an artifact of our sample. Deploying tags on adults and subadults in the same overwintering location could help determine whether the differences in haul‐out and emergence behavior observed in this study were due to habitat or age‐related differences in foraging efficiency and physiology. The environmental data used in our HMMs are coarse (25–32 km resolution) and may not fully represent the local conditions experienced by individual seals. Remotely sensed snow and sea ice products often have missing data along coastlines where ringed seals occur in higher densities, further obscuring fine‐scale, accurate measurements. Due to the uncertainty and sparseness of the ringed seal location data, there is also potential error in which spatial environmental covariate values (i.e., which cell of data) were assigned to haul‐out records. However, we expect this error to be minimal due to the high spatial autocorrelation of the environmental variables and their coarse spatial scale relative to ringed seal movements. Nonetheless, because we filled in missing seal locations to extract environmental covariates, the HMMs' estimates of uncertainty for covariate relationships may be biased low. As a result, the covariate relationships and confidence intervals shown in Figures 5 and 7 should be interpreted conservatively. Future studies using finer‐resolution environmental data could use multiple imputation or bilinear interpolation to account for potential location error when assigning covariate values (McClintock and Michelot 2018).

For the adult seals with two consecutive years of haul‐out data (*n* = 7), it was difficult to assess whether their individual behavior and emergence timing were consistent between years due to differences in temporal coverage (e.g., some tags provided data during only May–June one year, and only February–March the following year). However, there were four seals with some haul‐out data during May and/or June of consecutive years. One of these seals had a Viterbi‐estimated emergence date during its first year but not during its second year when it spent noticeably less time hauled out. Three seals showed more consistent behavior between consecutive years, but with interannual differences in timing that corresponded with interannual differences in temperature. During whichever year had earlier spring warming, the seals switched their haul‐out behavior earlier in the season and also had earlier Viterbi‐estimated emergence dates. Longitudinal data on individual ringed seals are sparse, and further tagging studies deploying SPOT tags (which have the potential to collect data over multiple years) would be valuable for investigating interannual variability in behavior.

We did not explore short‐term variability in haul‐out behavior in this study (i.e., effects of weather between or within days). In the future, our HMMs could be combined with short‐term haul‐out probability models (e.g., Hamilton et al. 2018) to address this source of variability in aerial survey counts. Our HMMs could also be expanded to include covariates on the state‐dependent probability distributions, for example, to allow weather to influence a seal's day‐to‐day haul‐out behavior within each behavioral state. Movement of animals is another factor that complicates aerial survey interpretation and is not addressed by our models (Carlens et al. 2006).

### 4.3 Implications for Aerial Surveys

Despite the complexities regarding seal movement and short‐term weather variability, the broader behavioral and phenological patterns identified in this study provide a framework for addressing availability bias in aerial surveys. With information on date, location, and records of air temperature and daylength during aerial surveys, our model results can be used to predict (a) what fraction of ringed seals are likely to be visible on the ice surface (versus in snow lairs or in the water), and (b) the typical haul‐out behavior of those seals during the survey window. For instance, results from the present analysis are currently being used to determine correction factors for ringed seals encountered during recent aerial surveys conducted in the Bering, Chukchi, and Beaufort seas (P. Conn, unpublished data). Furthermore, our results can directly inform aerial survey planning. The relationship we found between mean emergence date and latitude indicates that surveys in high‐latitude regions should be conducted later in the spring to align with peak ringed seal availability. By synchronizing survey timing with local emergence phenology, survey‐based population monitoring programs can maximize the proportion of seals available to be counted and minimize any bias associated with ringed seals hidden in lairs.

## 5 Conclusion

This study uses a novel method to estimate the timing of ringed seal emergence from haul‐out data provided by satellite tags. Our models identified that emergence probability for adult seals is associated with changes in air temperature and daylength, which we speculate is due to a combination of suitable conditions for molting and unsuitable conditions for continued lair use. We found that subadults may have a weaker seasonal pattern in haul‐out behavior than adults, potentially indicating less use of lairs, though this requires further research. These findings improve our understanding of ringed seal behavioral ecology during the pupping and molting periods, which are thought to be vulnerable to climate change. Haul‐out data from tagged ringed seals will continue to be valuable for monitoring the timing of emergence in a warming Arctic.

## Author Contributions

**Jessica M. Lindsay:** conceptualization (equal), data curation (equal), formal analysis (lead), visualization (lead), writing – original draft (lead), writing – review and editing (lead). **Paul B. Conn:** conceptualization (equal), supervision (equal), writing – review and editing (equal). **Peter L. Boveng:** conceptualization (supporting), data curation (equal), funding acquisition (equal), resources (equal), writing – review and editing (equal). **Justin A. Crawford:** data curation (equal), funding acquisition (equal), resources (equal), writing – review and editing (equal). **Lori T. Quakenbush:** data curation (equal), funding acquisition (equal), resources (equal), writing – review and editing (equal). **Andrew L. Von Duyke:** data curation (equal), funding acquisition (equal), resources (equal), writing – review and editing (equal). **Kristin L. Laidre:** conceptualization (supporting), supervision (equal), writing – review and editing (equal).

## Conflicts of Interest

The authors declare no conflicts of interest.

## Supporting information


**Data S1:** ece372948‐sup‐0001‐supinfo.docx.

## Data Availability

The cleaned ringed seal haul‐out data, daily estimated locations, environmental covariates, and model code are publicly available on GitHub at https://github.com/j‐m‐lindsay/RingedSealEmergenceRepo/tree/main.
