# Supplementary material for: Ringed Seal (Pusa hispida) Haul‐Out Behavior and Emergence Timing in the Bering, Chukchi, and Beaufort Seas
Source: Ecol Evol. 2026 Jan 25;16(1):e72948. doi: 10.1002/ece3.72948 (PMC12832195; doi:10.1002/ece3.72948)

# **Supplementary Tables and Figures**

Table S1. Summary of tag deployment details for each of the ringed seal tagging projects used in the present study. For deployment locations, the general areas where seals were tagged within each sea are noted in parentheses.

| **Reference for project details** | **Deployment years** | **Deployment months** | **Deployment locations** | **N total** |
| --- | --- | --- | --- | --- |
| Kelly et al. (2010) | 2005–2007 | March–May | Chukchi Sea (Kotzebue Sound, AK; Peard Bay, AK), & Beaufort Sea (Garry Island, Canada) | 23 |
| Crawford et al. (2012, 2018) | 2007–2010 | Sept.–Oct. | Chukchi Sea (Kotzebue Sound, AK) | 19 |
| Von Duyke et al. (2020, unpubl. data) | 2011–2018 | July | Beaufort Sea (Utqiaġvik, AK) | 13 |
| Quakenbush et al. (2019) | 2014–2019 | June | Chukchi Sea (Kotzebue Sound, AK) & Beaufort Sea (Utqiaġvik, AK) | 9 |

Table S2. Demographic, biometric, and tagging information for individual ringed seals (n = 64) included in the present study, and the years and date ranges within February to mid-June that the seal transmitted haul-out data. Tags were manufactured by Wildlife Computers and models include: ‘SPOT’ tags (permanently attached to a hind flipper), ‘SPLASH’ tags (glued to hair on the head or upper back), or ‘both’ tag models.

| Animal ID | Sex | Weight (kg) | Claw bands | Age class | Tag  model(s) | Tag position(s) | Capture date | Capture latitude (°N) | Capture longitude (°E) | Data year(s) | First day of haul-out data | Last day of haul-out data | Elapsed days |
| --- | --- | --- | --- | --- | --- | --- | --- | --- | --- | --- | --- | --- | --- |
| PH2005_5997 | F | 44 | 7 | Adult | SPOT | flipper | 1-May-2005 | 70.83 | -158.96 | 2005 | 2-May | 15-Jun | 45 |
| PH2005_5998 | F | 69 | 7 | Adult | SPOT | flipper | 4-May-2005 | 70.83 | -158.96 | 2005 | 5-May | 15-Jun | 43 |
| PH2005_5999 | F | 57 | 6 | Adult | SPOT | flipper | 6-May-2005 | 70.83 | -158.96 | 2005  2006 | 8-May  3-Feb | 15-Jun  12-Mar | 41  38 |
| PH2005_6000 | F | 59 | 4 | Adult | SPOT | flipper | 9-May-2005 | 70.83 | -158.96 | 2005 | 11-May | 15-Jun | 38 |
| PH2005_6001 | F | 51 | 8 | Adult | SPOT | flipper | 10-May-2005 | 70.83 | -158.96 | 2005 | 15-May | 15-Jun | 32 |
| PH2005_6002 | F | 53.5 | 7 | Adult | SPOT | flipper | 11-May-2005 | 70.83 | -158.96 | 2005 | 14-May | 15-Jun | 33 |
| PH2005_6003 | M | 57 | 7 | Adult | SPOT | flipper | 25-May-2005 | 70.83 | -158.96 | 2005  2006 | 26-May  6-Feb | 15-Jun  15-Jun | 22  130 |
| PH2005_6004 | M | 53 | 5 | Adult | SPOT | flipper | 16-May-2005 | 70.83 | -158.96 | 2005  2006 | 17-May  15-Feb | 15-Jun  15-Jun | 30  121 |
| PH2005_6005 | M | 31 | 6 | Adult | SPOT | flipper | 18-May-2005 | 70.83 | -158.96 | 2005  2006 | 19-May  1-Feb | 12-Jun  25-Feb | 26  25 |
| PH2005_6006 | F | 47 | 6 | Adult | SPOT | flipper | 19-May-2005 | 70.83 | -158.96 | 2005 | 21-May | 15-Jun | 28 |
| PH2005_6007 | M | 19 | ≥0 | Subadult | SPOT | flipper | 22-May-2005 | 70.83 | -158.96 | 2005 | 23-May | 15-Jun | 24 |
| PH2006_6009 | M | 55 | ≥5 | Adult | SPOT | flipper | 21-Mar-2006 | 69.49 | -135.7 | 2006 | 24-Mar | 26-Apr | 34 |
| PH2006_6010 | M | 54 | ≥6 | Adult | SPOT | flipper | 20-Mar-2006 | 69.49 | -135.7 | 2006 | 14-May | 15-Jun | 33 |
| PH2006_6011 | M | 66 | ≥5 | Adult | SPOT | flipper | 21-Mar-2006 | 69.49 | -135.7 | 2006 | 1-May | 15-Jun | 46 |
| PH2006_6012 | F | 74 | ≥6 | Adult | SPOT | flipper | 23-Mar-2006 | 69.49 | -135.7 | 2006 | 23-Mar | 15-Jun | 85 |
| PH2006_6013 | F | 58 | ≥6 | Adult | SPOT | flipper | 23-Mar-2006 | 69.49 | -135.7 | 2006 | 15-May | 7-Jun | 24 |
| PH2006_6014 | F | 64 | ≥5 | Adult | SPOT | flipper | 25-Mar-2006 | 69.49 | -135.7 | 2006 | 5-May | 15-Jun | 42 |
| PH2006_6015 | M | 64 | 7 | Adult | SPOT | flipper | 28-Apr-2006 | 70.83 | -158.96 | 2006 | 29-Apr | 15-Jun | 48 |
| PH2006_6016 | M | 60 | 6 | Adult | SPOT | flipper | 2-May-2006 | 70.83 | -158.96 | 2006  2007 | 8-May  2-Feb | 13-Jun  5-Jun | 37  124 |
| PH2006_6018 | M | 59 | ≥6 | Adult | SPOT | flipper | 14-May-2006 | 70.83 | -158.96 | 2006 | 15-May | 13-Jun | 31 |
| PH2006_6019 | F | 53 | 7 | Adult | SPOT | flipper | 14-May-2006 | 70.83 | -158.96 | 2006 | 15-May | 15-Jun | 33 |
| PH2007_6020 | M | 60.8 | - | Adult | SPOT | flipper | 17-May-2007 | 70.83 | -158.96 | 2007 | 18-May | 12-Jun | 26 |
| PH2007_6021 | M | - | ≥4 | Subadult | SPOT | flipper | 23-May-2007 | 70.83 | -158.96 | 2007 | 24-May | 15-Jun | 23 |
| PH2011BW02 | M | 30.4 | ≥5 | Adult | SPOT | flipper | 14-Jul-2011 | 71.29 | -156.8 | 2012 | 17-May | 10-Jun | 25 |
| PH2011BW03 | M | 24.8 | ≥4 | Subadult | Both | head, flipper | 15-Jul-2011 | 71.29 | -156.8 | 2012 | 2-Feb | 4-Jun | 124 |
| PH2011BW10 | F | 26.6 | 8 | Adult | SPLASH | head | 20-Jul-2011 | 71.29 | -156.8 | 2012 | 5-Feb | 30-Apr | 86 |
| PH2011BW11 | F | 23.2 | 7 | Adult | SPLASH | head | 21-Jul-2011 | 71.29 | -156.8 | 2012 | 5-Feb | 1-Jun | 118 |
| PH2011BW12 | M | 27.2 | 8 | Adult | SPLASH | head | 21-Jul-2011 | 71.29 | -156.8 | 2012 | 1-Feb | 3-May | 93 |
| PH2014BW02 | M | 18.3 | - | Subadult | SPLASH | head | 22-Jul-2014 | 71.29 | -156.8 | 2015 | 1-Feb | 2-Feb | 2 |
| PH2016BW01 | M | 50.9 | ≥6 | Adult | Both | head, flipper | 1-Jul-2016 | 71.29 | -156.8 | 2017 | 1-Feb | 4-Jun | 124 |
| PH2016BW04 | M | 49.1 | ≥6 | Adult | Both | head, flipper | 1-Jul-2016 | 71.29 | -156.8 | 2017 | 1-Feb | 18-Mar | 46 |
| PH2016BW09 | F | 46.7 | ≥5 | Adult | SPLASH | back | 2-Jul-2016 | 71.29 | -156.8 | 2017 | 7-Feb | 22-Feb | 16 |
| PH2016BW10 | F | 40 | ≥4 | Adult | SPLASH | back | 2-Jul-2016 | 71.29 | -156.8 | 2017 | 1-Feb | 9-Feb | 9 |
| PH2016BW11 | M | 36.6 | ≥5 | Adult | SPLASH | head | 2-Jul-2016 | 71.29 | -156.8 | 2017 | 1-Feb | 24-Feb | 24 |
| PH2017BW01 | M | 30.8 | ≥5 | Adult | Both | back, flipper | 3-Jul-2017 | 71.29 | -156.8 | 2018 | 1-Feb | 20-Mar | 48 |
| PH2018BW01 | F | 20 | 0 | Subadult | SPLASH | back | 9-Jul-2018 | 71.29 | -156.8 | 2019 | 3-Feb | 17-Feb | 15 |
| PH2019_9001 | M | 57.8 | 6 | Adult | SPOT | flipper | 24-Jun-2019 | 71.29 | -156.6 | 2020  2021 | 2-Feb  7-Mar | 14-Jun  3-Apr | 134  28 |
| RS07-04-F | F | 47.7 | - | Adult | SPLASH | back | 14-Oct-2007 | 66.99 | -162.84 | 2008 | 1-Feb | 14-May | 104 |
| RS07-06-M | M | 51.8 | - | Adult | SPLASH | back | 25-Oct-2007 | 66.99 | -162.84 | 2008 | 7-Feb | 16-Feb | 10 |
| RS07-10-F | F | 29.1 | - | Subadult | SPOT | flipper | 21-Oct-2007 | 66.99 | -162.84 | 2008 | 12-Mar | 12-Jun | 93 |
| RS07-12-F | F | 38.2 | - | Adult | SPOT | flipper | 25-Oct-2007 | 66.99 | -162.84 | 2008 | 26-Feb | 11-Jun | 107 |
| RS07-14-M | M | 32.7 | - | Subadult | SPLASH | back | 21-Oct-2007 | 66.99 | -162.84 | 2008 | 1-Feb | 13-Jun | 134 |
| RS07-16-M | M | 63.6 | - | Adult | SPLASH | back | 25-Oct-2007 | 66.99 | -162.84 | 2008 | 3-Feb | 16-Feb | 14 |
| RS08-02-F | F | 42.7 | 5 | Adult | SPLASH | back | 12-Oct-2008 | 66.99 | -162.84 | 2009 | 1-Feb | 15-Jun | 135 |
| RS08-03-M | M | 47.3 | 7 | Adult | SPLASH | back | 19-Oct-2008 | 66.99 | -162.84 | 2009 | 2-Feb | 17-Feb | 16 |
| RS08-05-M | M | 24.5 | - | Subadult | SPLASH | back | 19-Oct-2008 | 66.99 | -162.84 | 2009 | 1-Feb | 14-May | 103 |
| RS08-06-M | M | 22.7 | - | Subadult | SPLASH | back | 20-Oct-2008 | 66.99 | -162.84 | 2009 | 1-Feb | 29-May | 118 |
| RS08-07-F | F | 25.4 | - | Subadult | SPLASH | back | 17-Oct-2008 | 66.99 | -162.84 | 2009 | 1-Feb | 6-Mar | 34 |
| RS08-08-M | M | 24.5 | - | Subadult | SPLASH | back | 19-Oct-2008 | 66.99 | -162.84 | 2009 | 2-Feb | 15-Jun | 134 |
| RS08-10-M | M | 21.4 | - | Subadult | SPLASH | back | 20-Oct-2008 | 66.99 | -162.84 | 2009 | 1-Feb | 14-Jun | 134 |
| RS08-11-M | M | 30 | - | Subadult | SPLASH | back | 14-Oct-2008 | 66.99 | -162.84 | 2009 | 1-Feb | 15-Jun | 135 |
| RS08-12-M | M | 45.5 | 6 | Adult | SPLASH | back | 20-Oct-2008 | 66.99 | -162.84 | 2009 | 6-Feb | 20-Feb | 15 |
| RS08-13-F | F | 23.2 | - | Subadult | SPLASH | back | 21-Oct-2008 | 66.99 | -162.84 | 2009 | 1-Feb | 15-Jun | 135 |
| RS09-01-M | M | 24.1 | - | Subadult | SPLASH | back | 26-Sep-2009 | 66.99 | -162.84 | 2010 | 1-Feb | 13-Jun | 133 |
| RS09-04-F | F | 22.7 | - | Subadult | SPLASH | back | 30-Sep-2009 | 66.99 | -162.84 | 2010 | 1-Feb | 15-Jun | 135 |
| RS09-08-F | F | 42.3 | - | Adult | SPLASH | back | 30-Sep-2009 | 66.99 | -162.84 | 2010 | 9-Feb | 2-Mar | 22 |
| RS14-01-M | M | 50.4 | 7 | Adult | SPOT | flipper | 18-Jun-2014 | 66.28 | -161.84 | 2015  2016 | 22-Feb  17-Feb | 29-May  9-May | 97  83 |
| RS14-02-F | F | 44 | 6 | Adult | Both | back, flipper | 18-Jun-2014 | 66.28 | -161.84 | 2015 | 16-Feb | 10-Jun | 115 |
| RS14-03-M | M | 52.8 | 6 | Adult | Both | back, flipper | 19-Jun-2014 | 66.28 | -161.84 | 2015 | 23-Feb | 12-Jun | 110 |
| RS14-04-F | F | 44.5 | 6 | Adult | SPOT | flipper | 19-Jun-2014 | 66.28 | -161.84 | 2015 | 5-Mar | 11-Jun | 99 |
| RS17-01-M | M | - | 7 | Adult | SPOT | flipper | 20-Jun-2017 | 66.26 | -161.53 | 2018 | 17-Feb | 15-May | 88 |
| RS19-02-M | M | 42.6 | 6 | Adult | SPOT | flipper | 22-Jun-2019 | 71.29 | -156.81 | 2020 | 27-Feb | 13-Jun | 108 |
| RS19-03-M | M | 38.3 | 10 | Adult | SPOT | flipper | 23-Jun-2019 | 71.29 | -156.81 | 2020 | 24-Mar | 14-Jun | 83 |
| RS19-04-M | M | 49 | 7 | Adult | SPOT | flipper | 23-Jun-2019 | 71.29 | -156.81 | 2020 | 2-Feb | 14-Jun | 134 |

Table S3. State-dependent probability distribution estimates of daily proportion hauled out and peak haul-out hour during the lair and emerged behavioral states, estimated by the best hidden Markov models for adult and subadult ringed seals. The standard error (SE) for each parameter estimate is given in parentheses.

|  |  | **Adult Model** | |  | **Subadult Model** | |  |
| --- | --- | --- | --- | --- | --- | --- | --- |
|  |  | **Lair state** | **Emerged State** |  | **Lair State** | **Emerged State** |  |
| **Observed variable** | **Distribution parameter** | **Estimate (SE)** | **Estimate (SE)** |  | **Estimate (SE)** | **Estimate (SE)** |  |
| Daily proportion hauled out | Shape1 | 1.393 (0.086) | 1.676 (0.093) |  | 1.462 (0.084) | 1.490 (0.205) |  |
|  | Shape2 | 3.220 (0.220) | 1.344 (0.069) |  | 4.422 (0.286) | 1.269 (0.168) |  |
|  | Zero-mass | 0.389 (0.018) | 0.006 (0.003) |  | 0.481 (0.016) | 0.108 (0.030) |  |
|  | One-mass | 0.005 (0.003) | 0.047 (0.008) |  | <0.001 (<0.001) | 0.026 (0.015) |  |
|  |  |  |  |  |  |  |  |
| Peak haul-out hour (radians) | Mean | 2.827 (0.156) | -0.015 (0.035) |  | 2.814 (0.136) | 0.210 (0.126) |  |
|  | Concentration | 0.441 (0.075) | 1.907 (0.096) |  | 0.472 (0.066) | 1.249 (0.201) |  |

Table S4: Estimated transition probabilities and regression coefficients from the best hidden Markov models for adult and subadult ringed seals. State transition probabilities are based on the mean values of any covariates (i.e., at the mean values of daylength and daily air temperature for the adult model).

|  | **Adult Model** | **Subadult Model** |
| --- | --- | --- |
| **State transition** | **Probability (SE)** | **Probability (SE)** |
| Lair 🡪 Lair | 0.9907 (0.004) | 0.9996 (0.001) |
| Lair 🡪 Emerged | 0.0093 (0.004) | 0.0004 (0.001) |
|  |  |  |
| **Regression coefficients** | **Estimate (SE)** | **Estimate (SE)** |
| Intercept | -7.385 (1.339) | -19.533 (4.138) |
| Daylength | 0.238 (0.059) | 0.841 (0.214) |
| Daily air temperature | 0.135 (0.074) | NA |


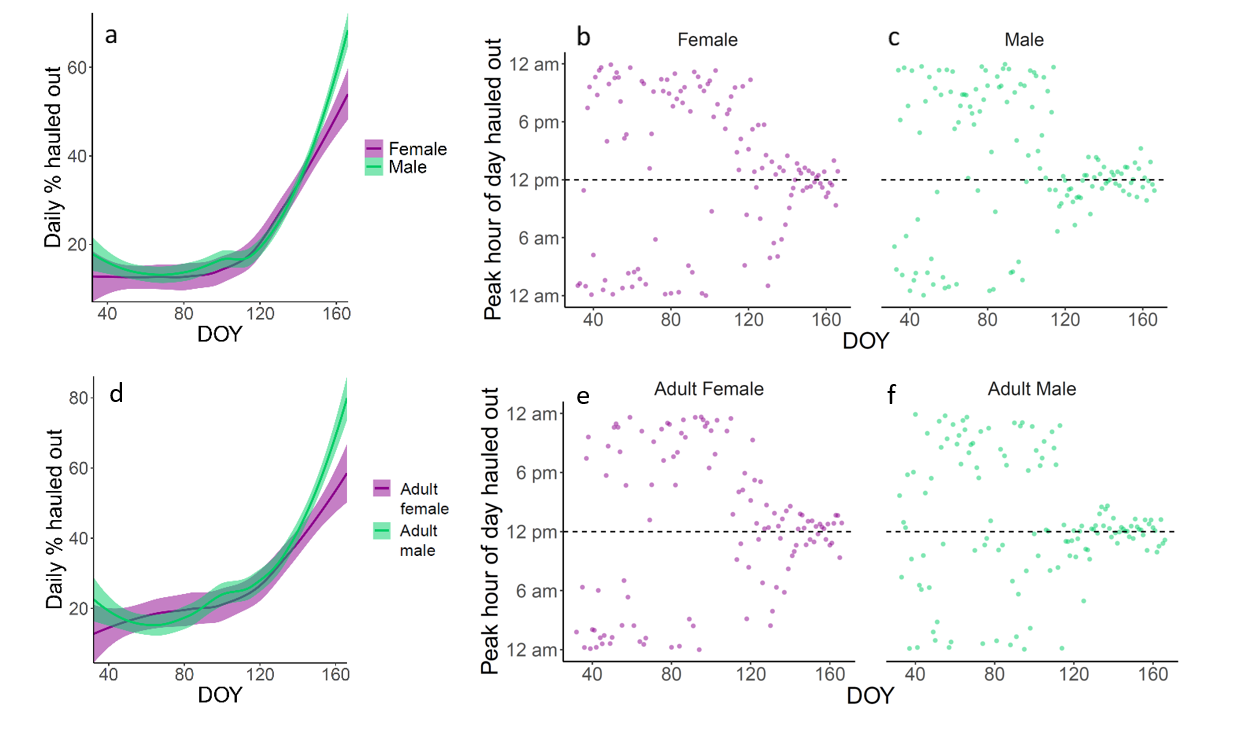


Figure S1. Exploratory plots of seasonal patterns in haul-out behavior by sex for combined age classes (a-c; n = 27 females, n = 44 males) and for adults only (d-f; n = 22 adult females, n = 33 males). Loess smooth and 95% confidence interval for mean daily % hauled out taken across all individuals within each sex (a,d). Mean peak haul-out hour taken across all individual females (b,e) or males (c,f). Peak hour of day hauled out is scaled to solar time, such that 12 pm represents solar noon.

Figure S2. Haul-out data and estimated behavioral states (lair and emerged) for individual adults, as estimated by the top HMM, which included daily air temperature + daylength as covariates on emergence probability. Each row represents summary plots for individual seals. If the Viterbi-decoded states indicated that an individual transitioned from the lair state to the emerged state, the day of year (DOY) of that transition is provided under the seal ID. NA indicates that the model did not identify an emergence date for that seal. The left and center plots show the daily proportion hauled out and peak haul-out hour, respectively, by DOY. Shading indicates the range of dates over which each seal was estimated to have been in the lair state (blue) or the emerged state (orange) by the Viterbi algorithm, which identifies a specific emergence date based on the optimal state sequence across a seal’s entire time series. The rightmost plots show the marginal probability of the seal being in the emerged state (black line) at each daily timestep and the DOY at which that probability exceeds 0.5 (vertical dashed line). This date generally aligns with the Viterbi-estimated emergence date, but differences can occur when seal behavior is ambiguous or when there are substantial gaps in a seal’s haul-out record. In these cases, the more gradual slope of the marginal probability line indicates greater uncertainty in emergence timing.


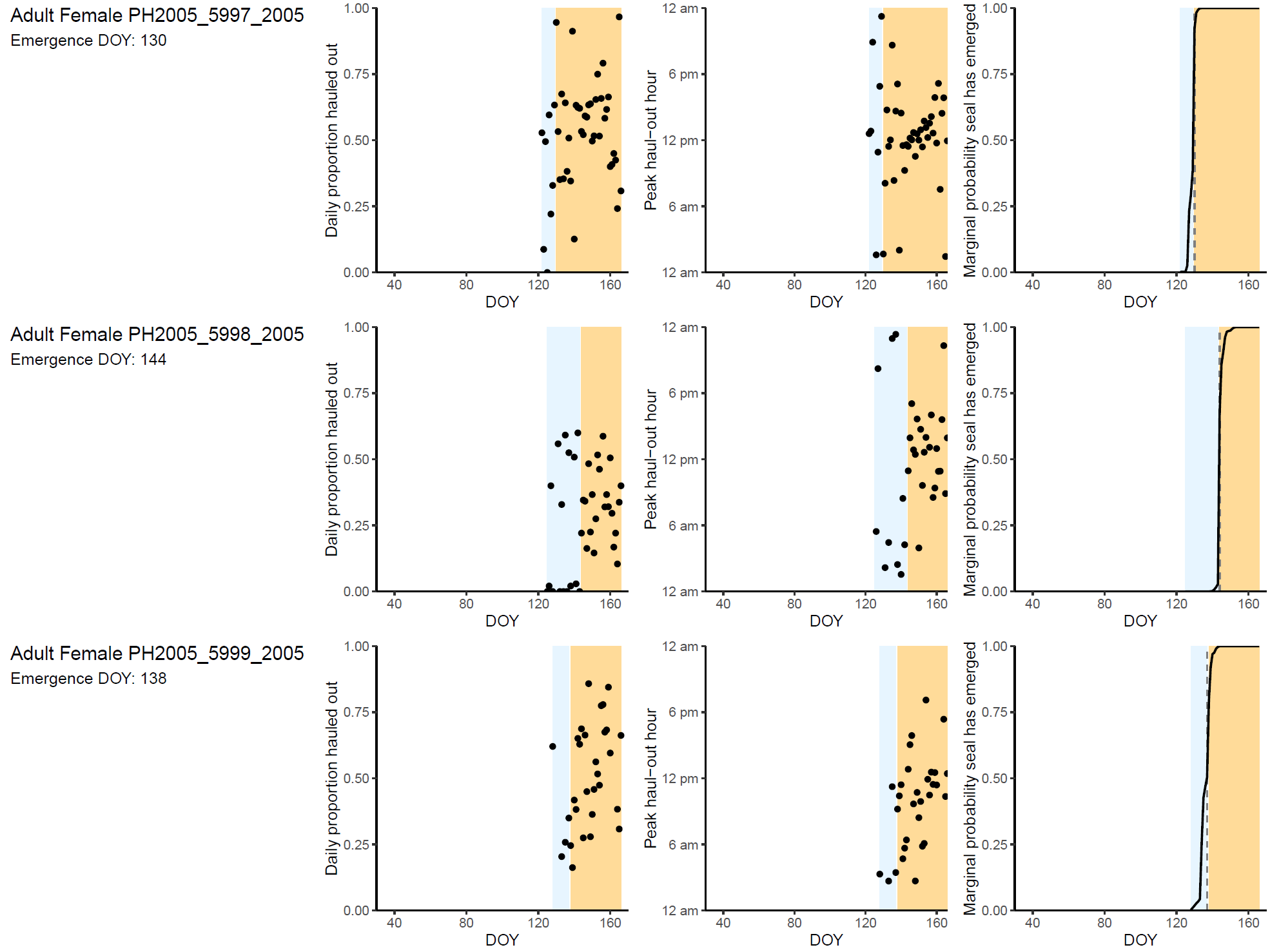


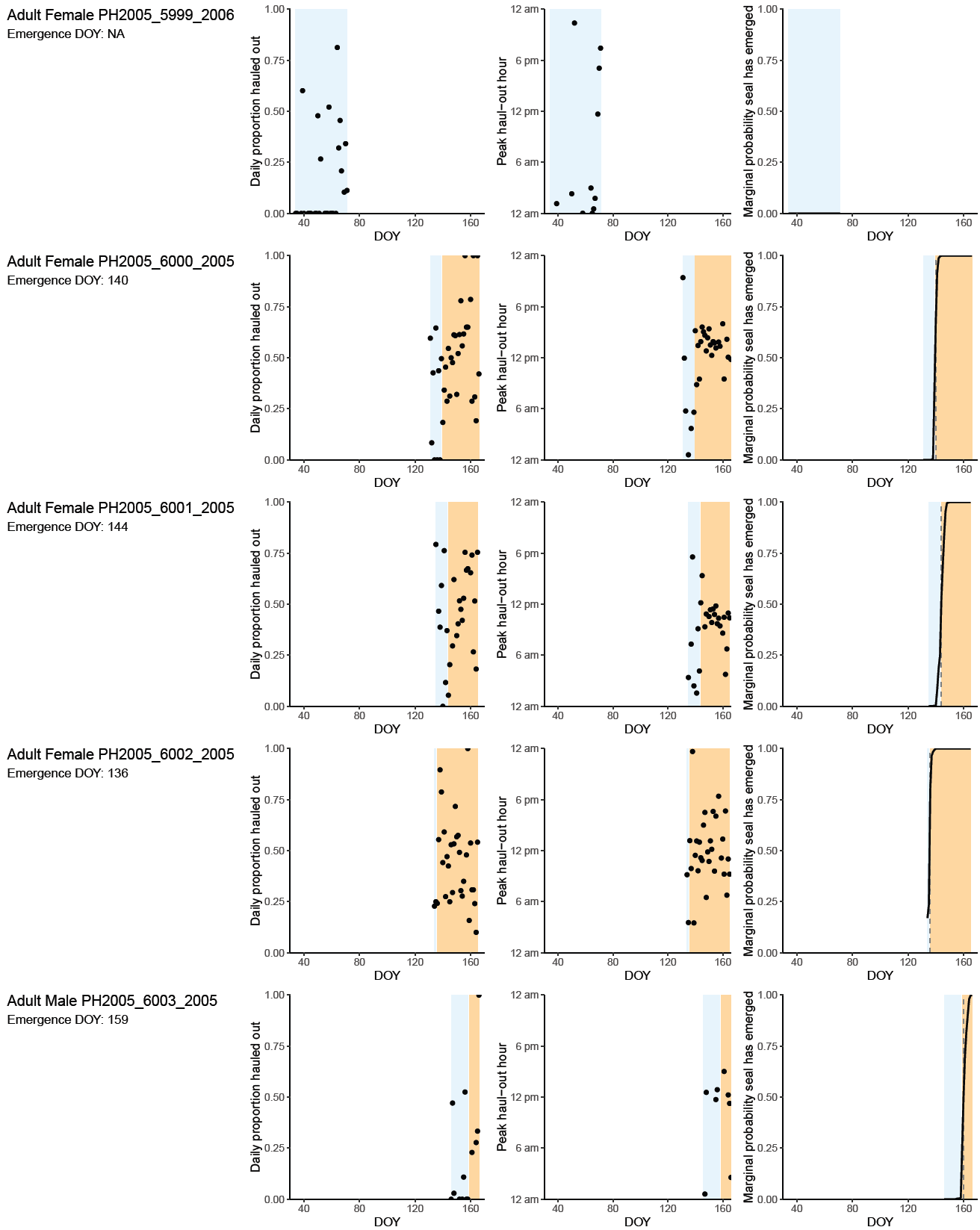


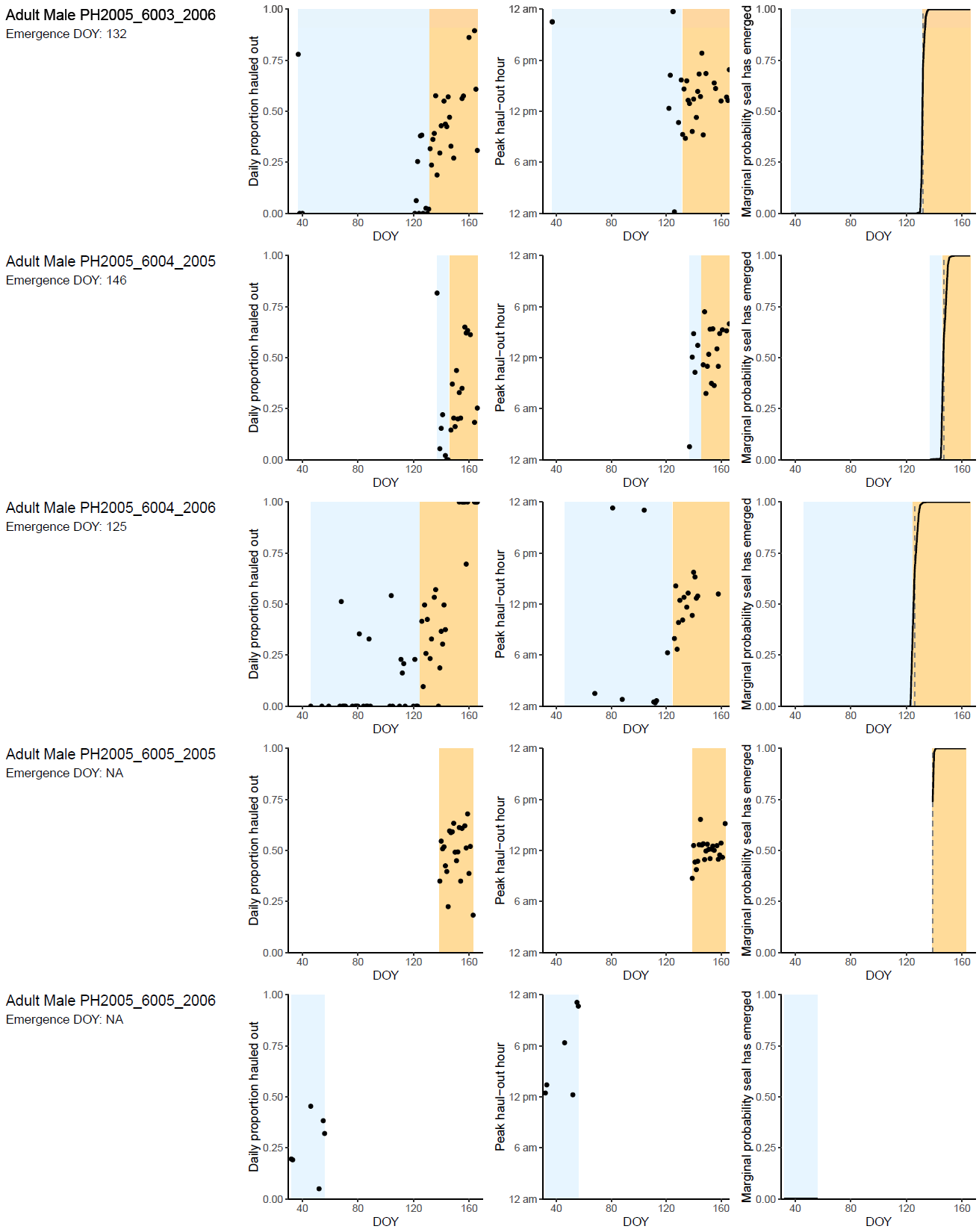


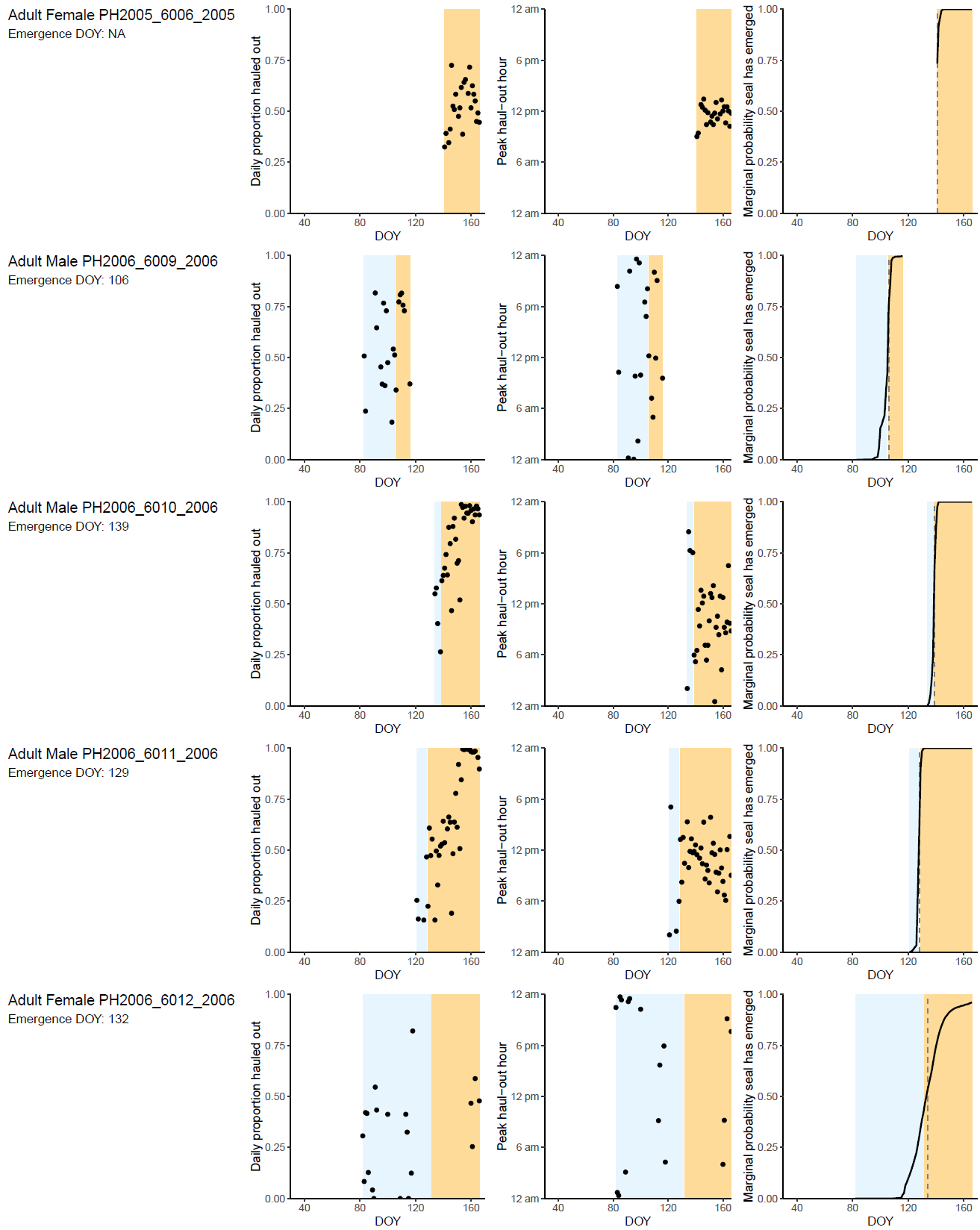


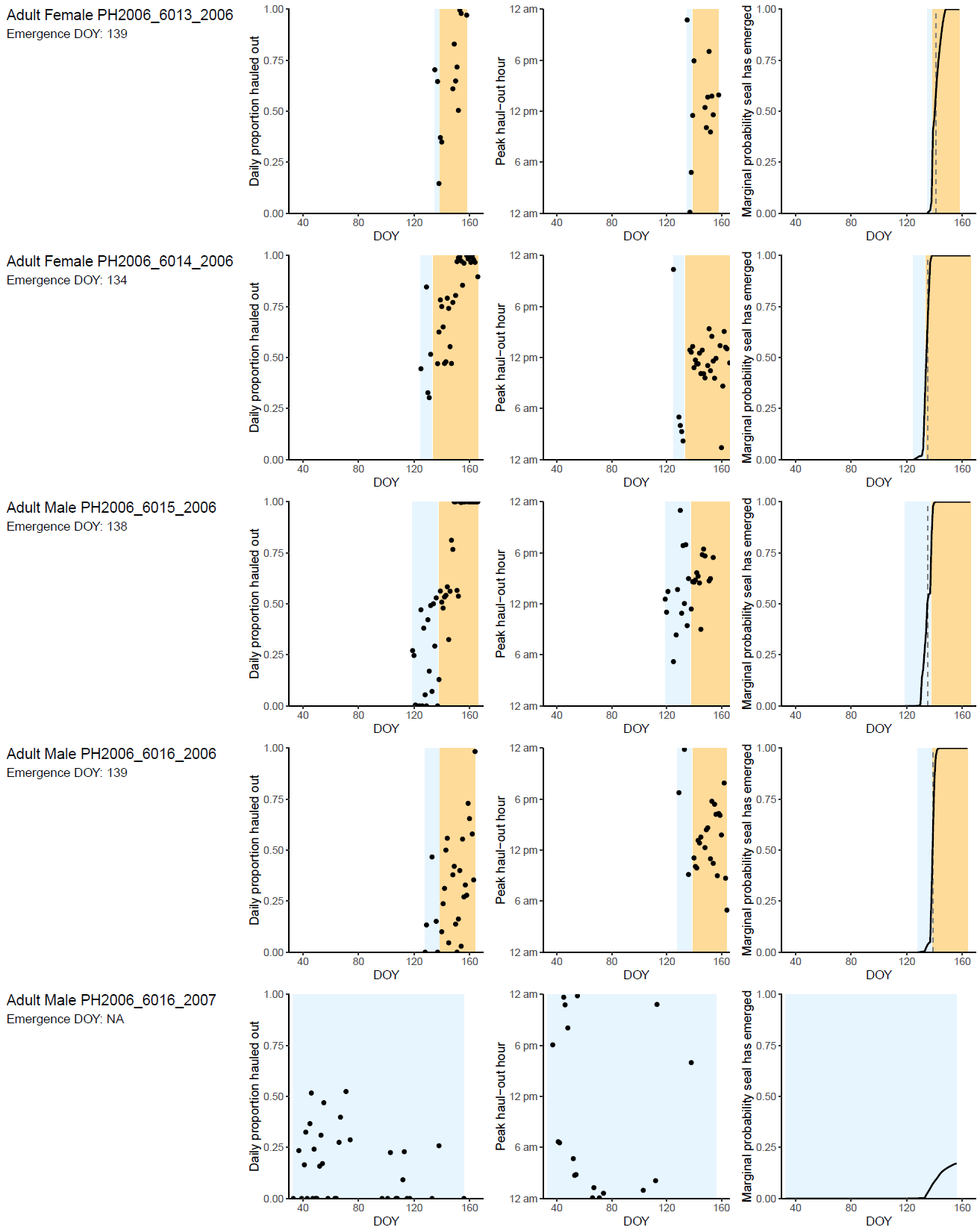

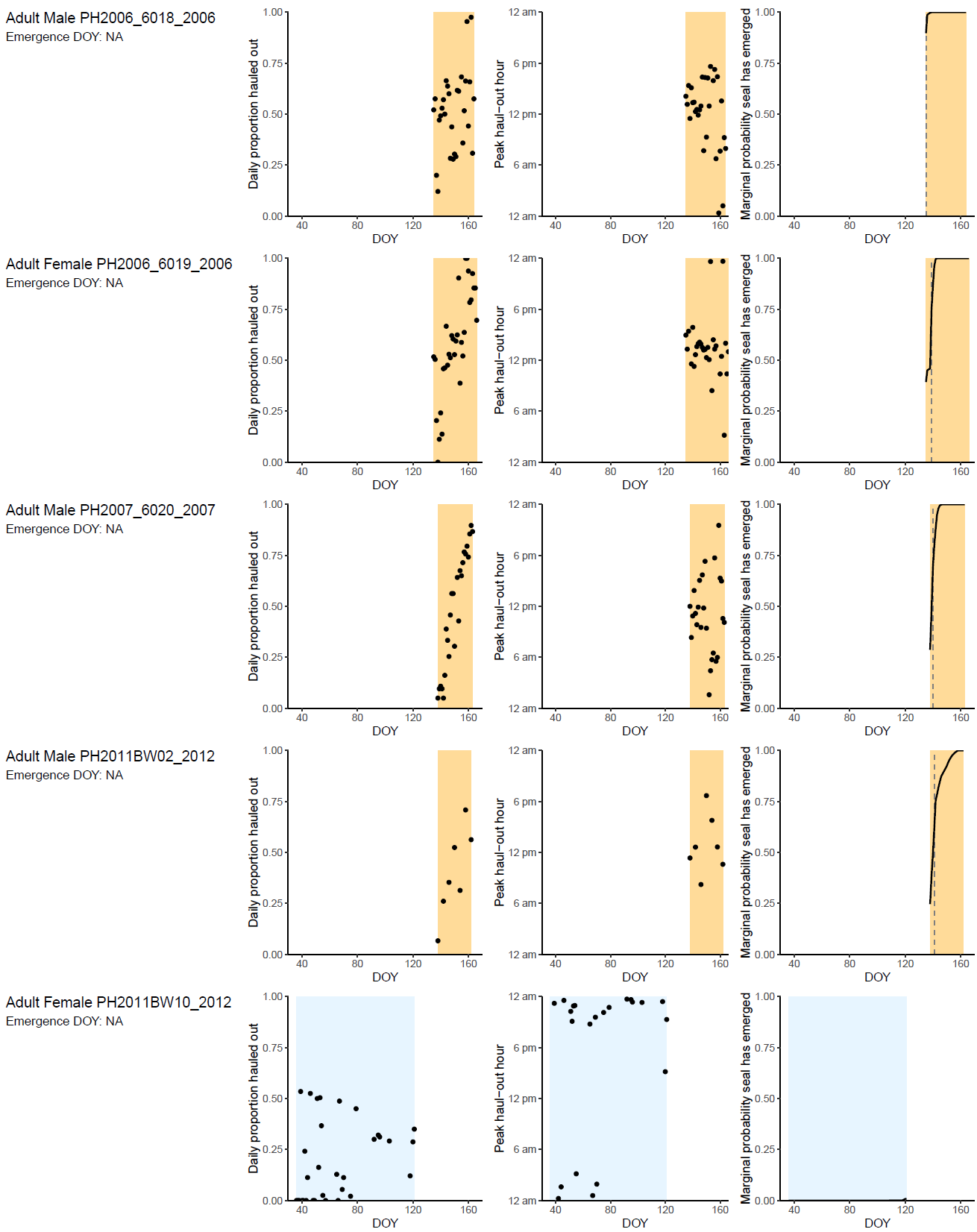

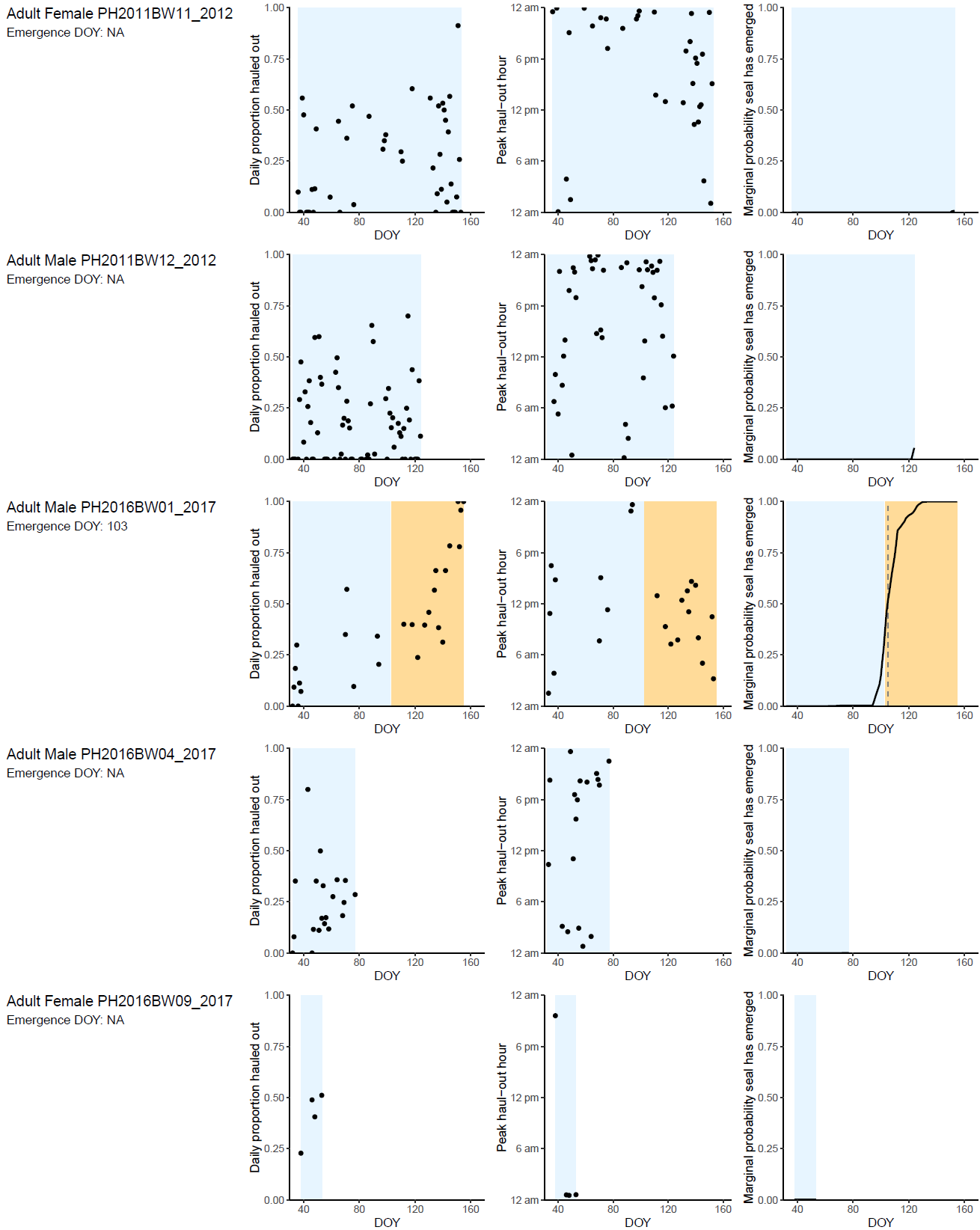

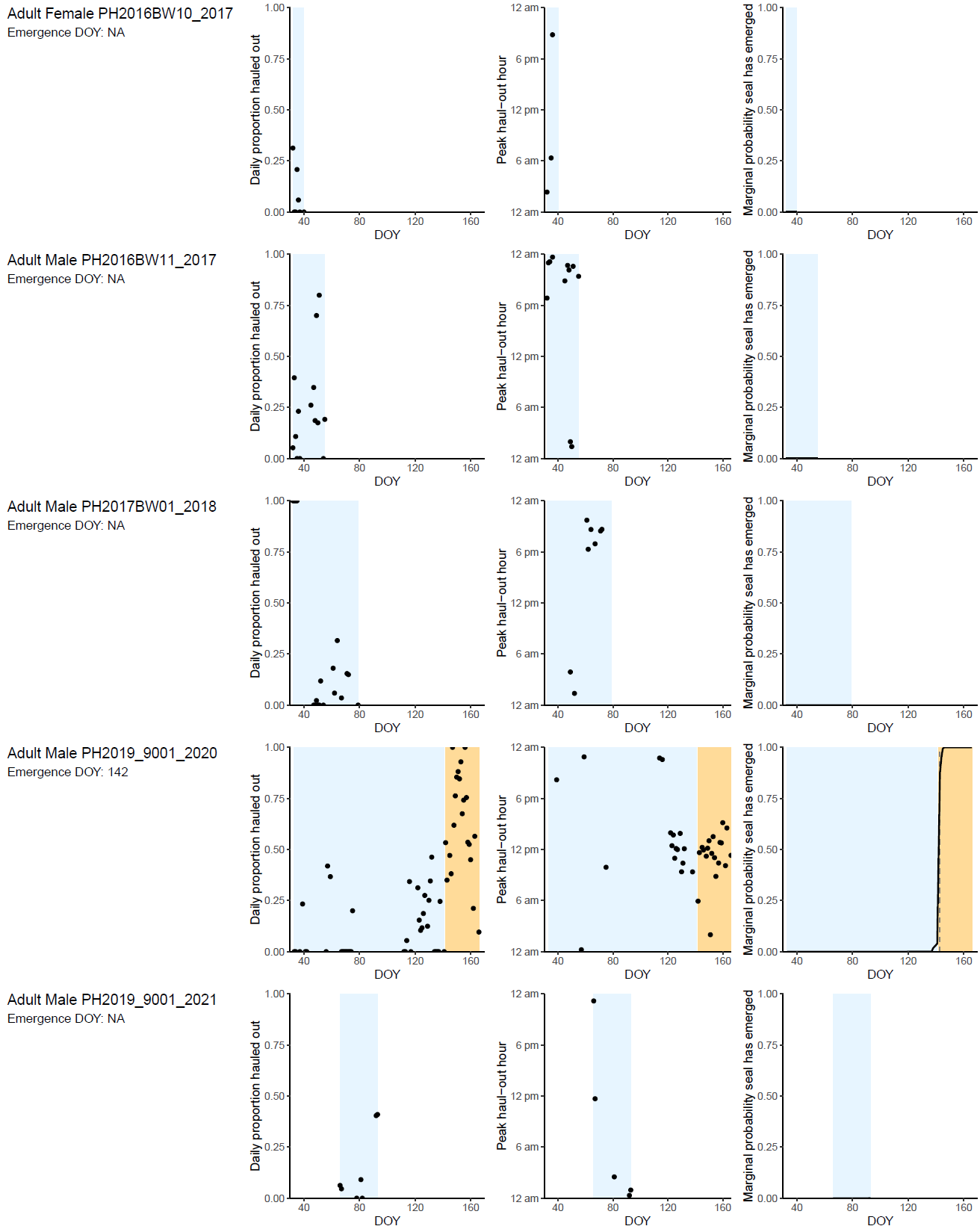

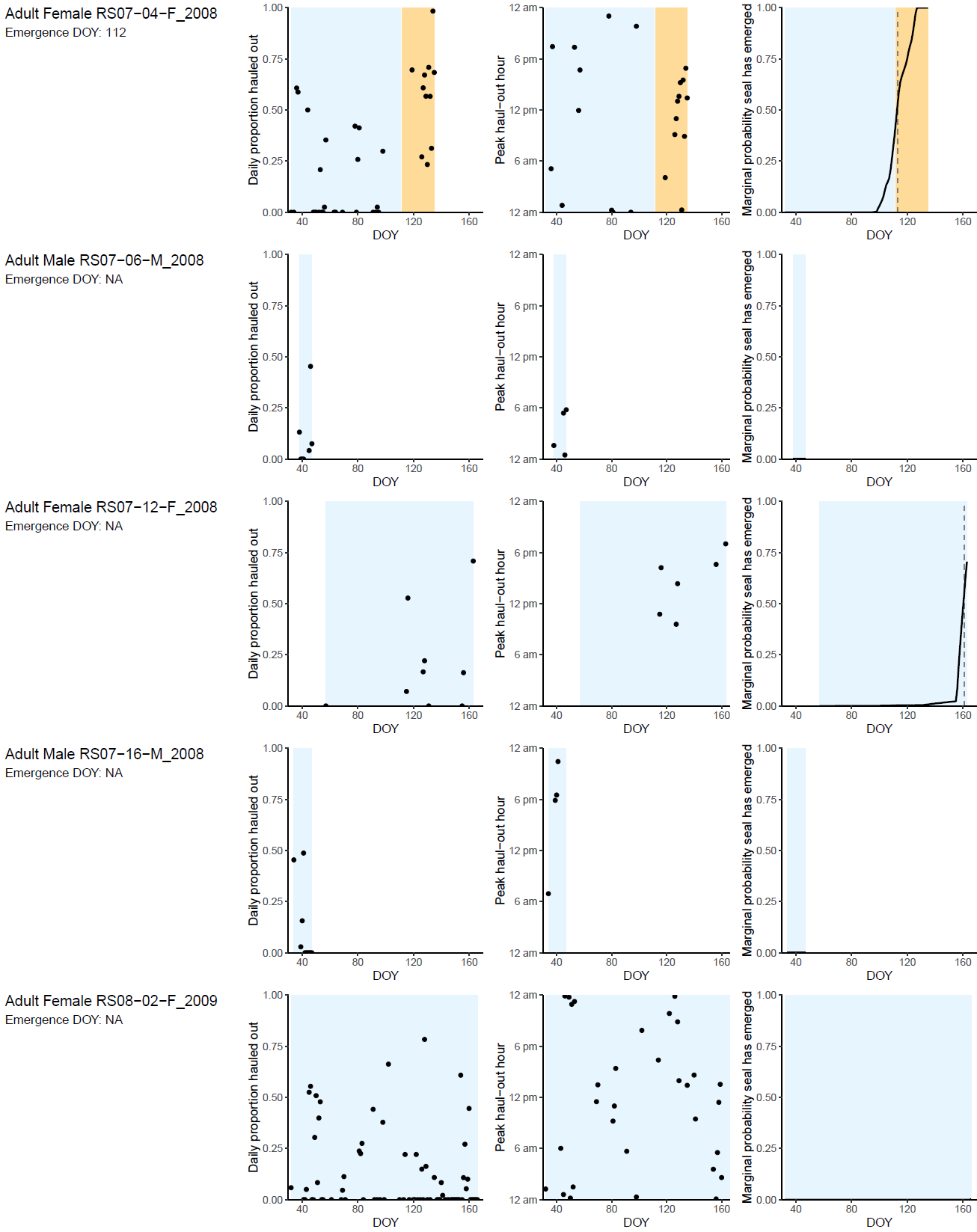

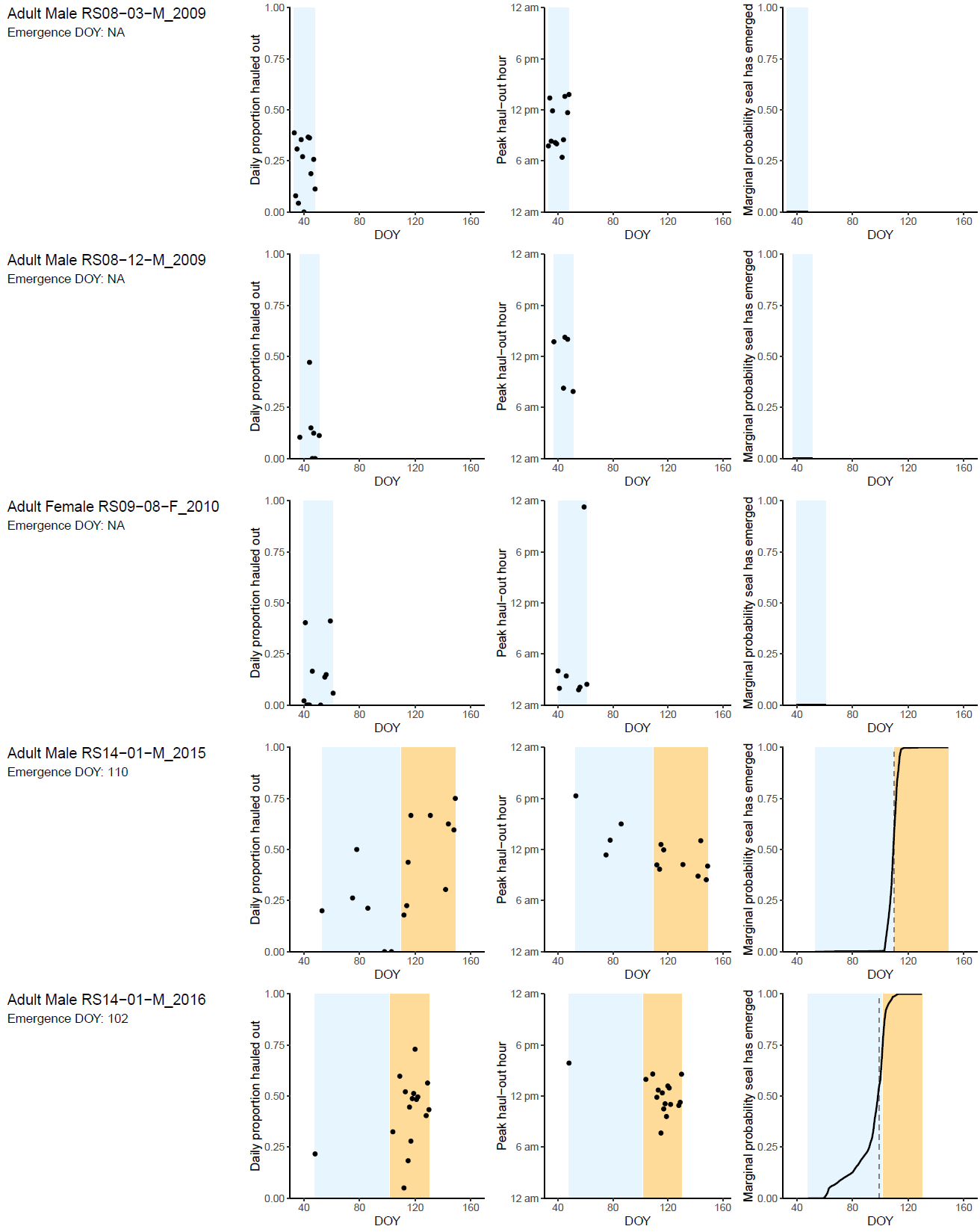

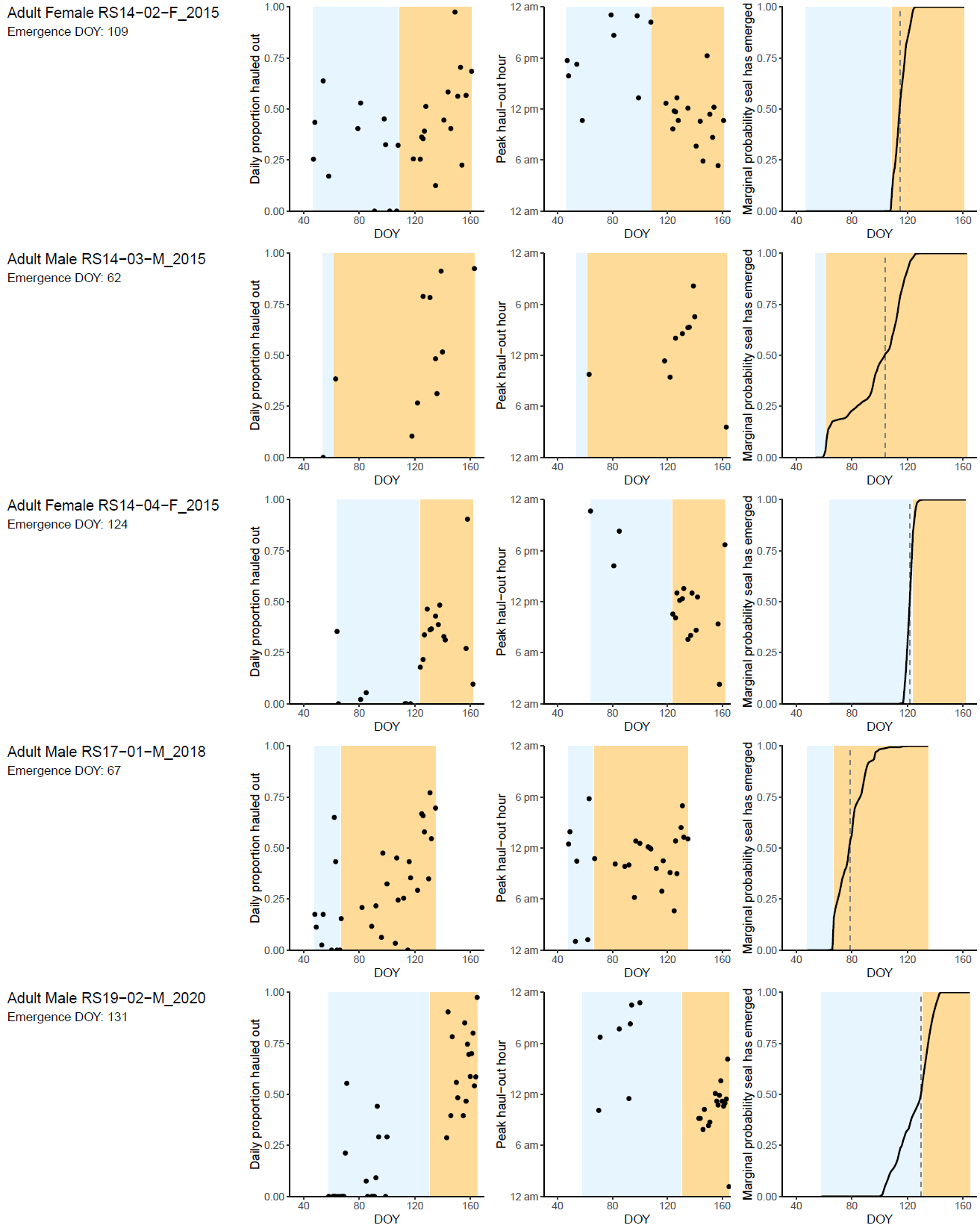

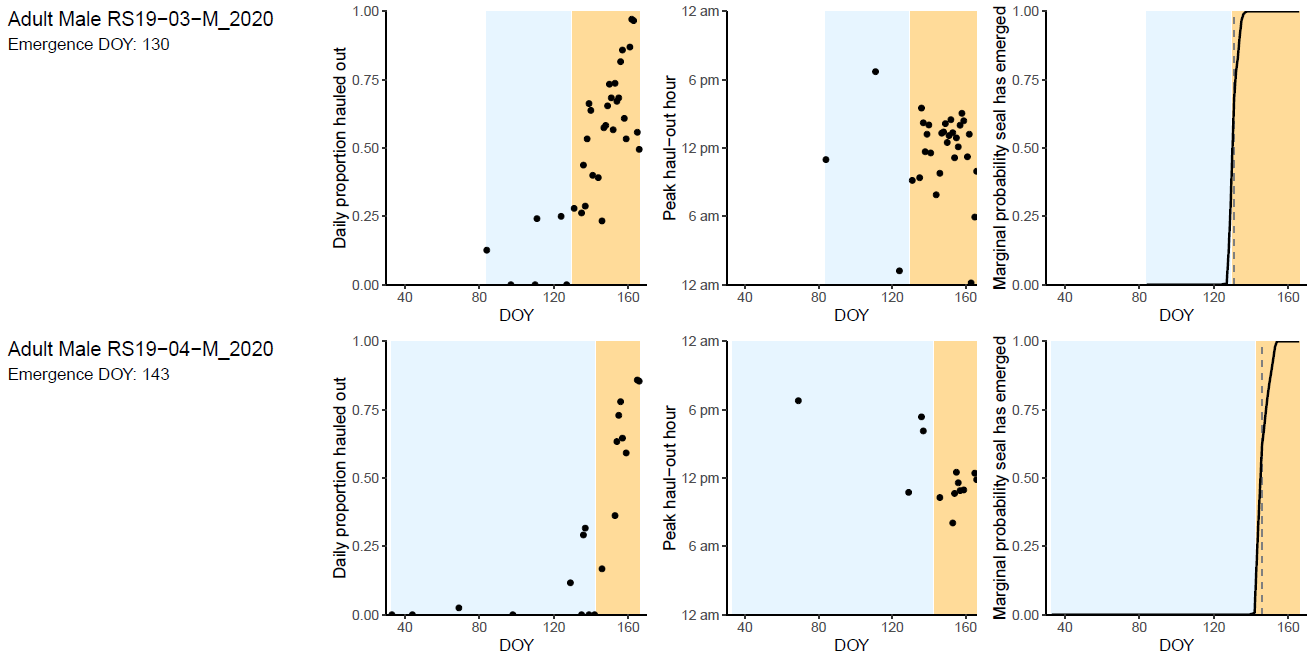


Figure S3. Haul-out data and estimated behavioral states (lair and emerged) for individual subadults, as estimated by the top HMM, which included daylength as a covariate on emergence probability. Each row represents summary plots for individual seals. If the Viterbi-decoded states indicated that an individual transitioned from the lair state to the emerged state, the day of year (DOY) of that transition is provided under the seal ID. NA indicates that the model did not identify an emergence date for that seal. The left and center plots show the daily proportion hauled out and peak haul-out hour, respectively, by DOY. Shading indicates the range of dates over which each seal was estimated to have been in the lair state (blue) or the emerged state (orange) by the Viterbi algorithm, which identifies a specific emergence date based on the optimal state sequence across a seal’s entire time series. The rightmost plots show the marginal probability of the seal being in the emerged state (black line) at each daily timestep and the DOY at which that probability exceeds 0.5 (vertical dashed line). This date generally aligns with the Viterbi-estimated emergence date, but differences can occur when seal behavior is ambiguous or when there are substantial gaps in a seal’s haul-out record. In these cases, the more gradual slope of the marginal probability line indicates greater uncertainty in emergence timing.


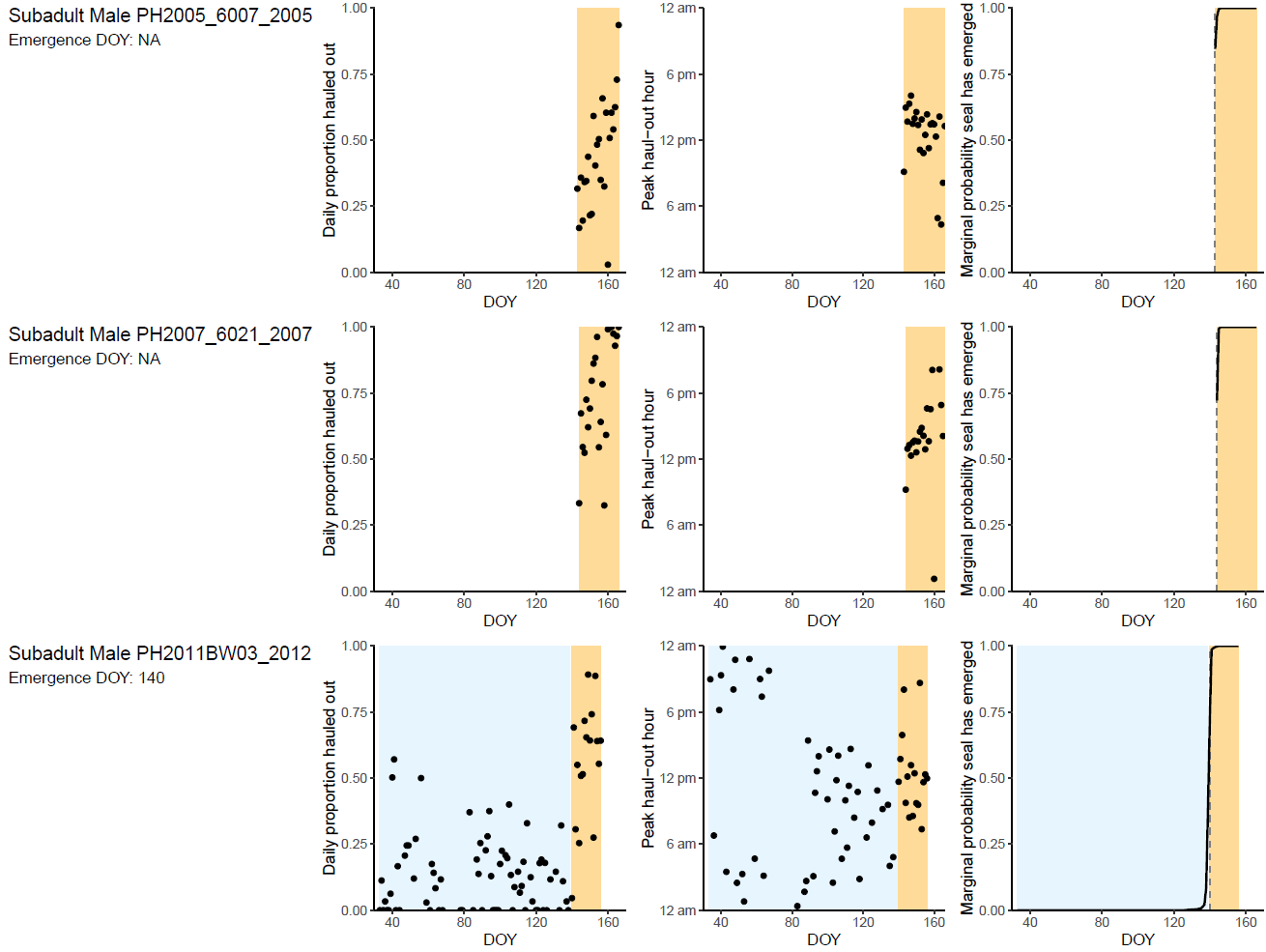


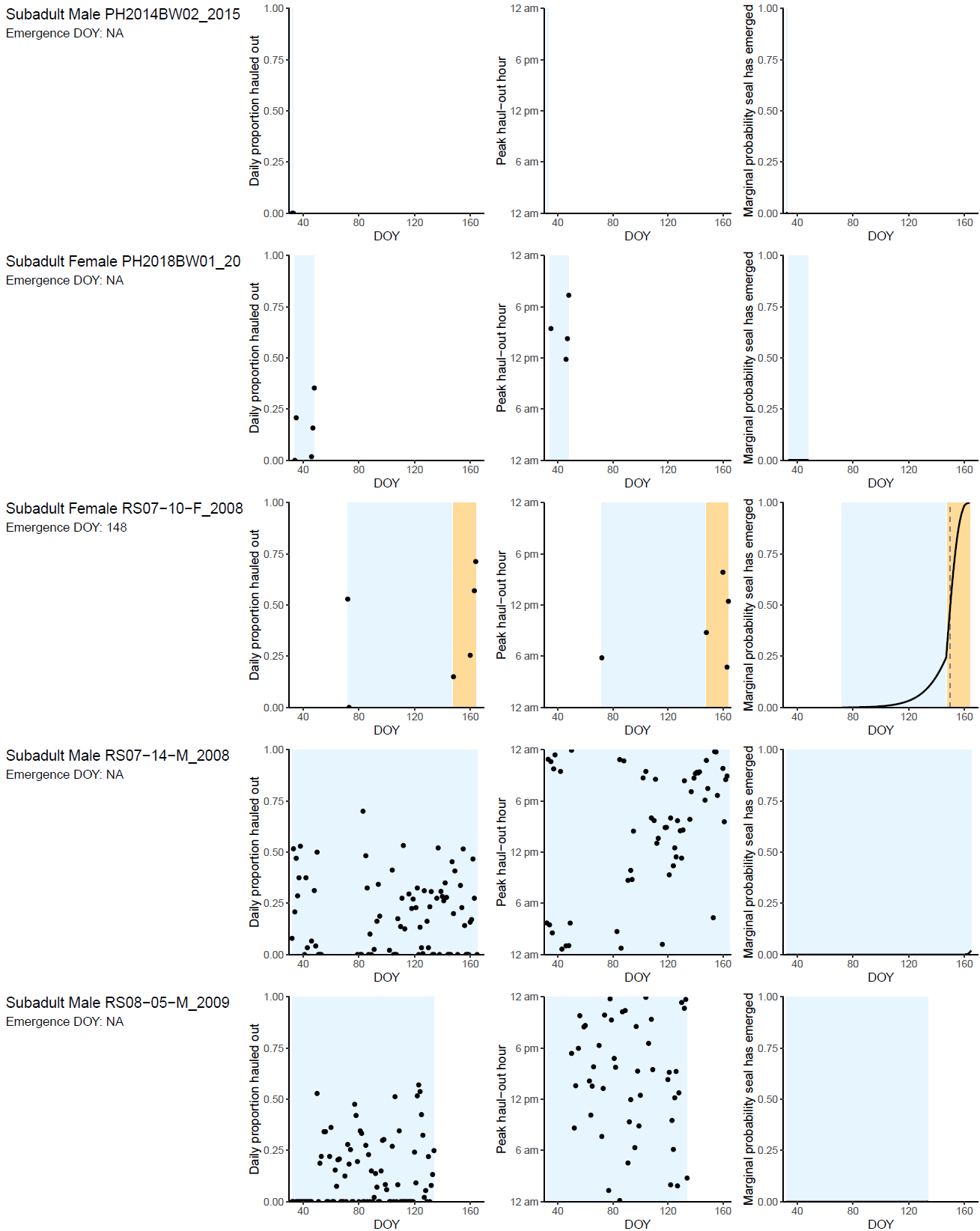

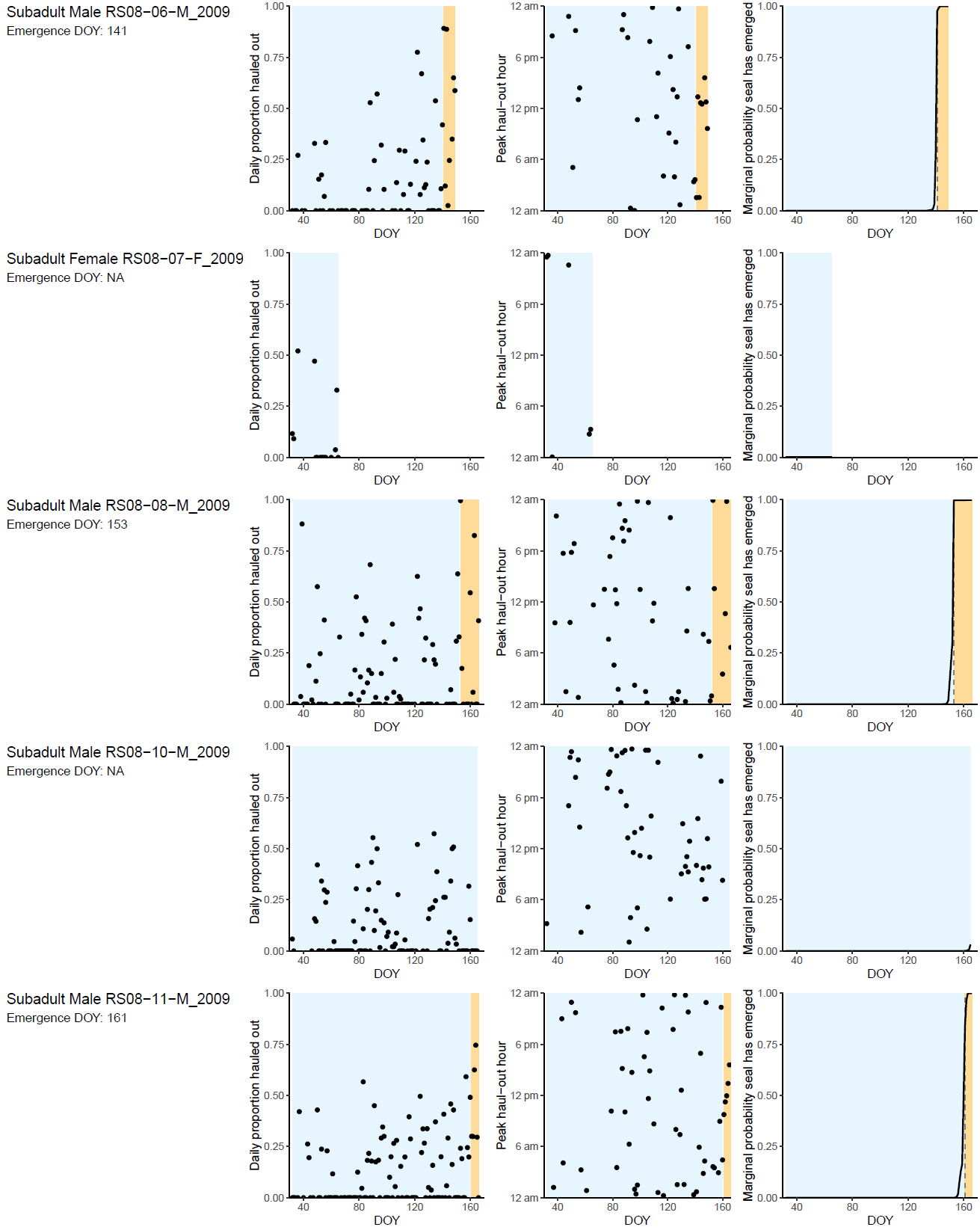

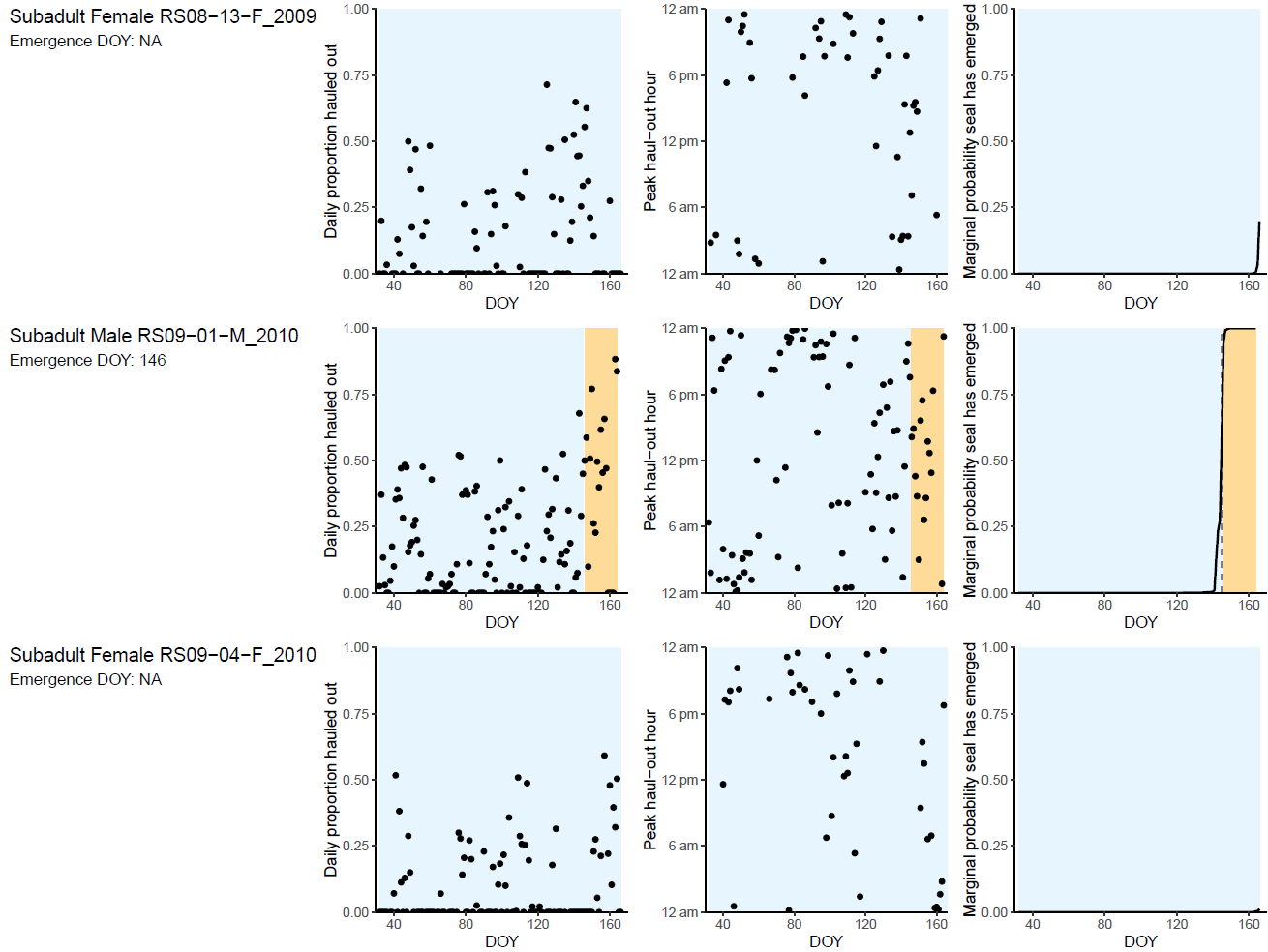


Figure S4. Viterbi-estimated emergence dates for individual adults over all leave-one-out iterations, where the best HMM for adult seals was re-fitted with one seal sequentially removed from the dataset. Two adults (PH2006_6012_2006 and PH2006_6019_2006) did not have Viterbi-estimated emergence dates when the HMM was fitted with the full dataset of adult seals (i.e., the model presented in the main text) but did have estimated emergence dates in some leave-one-out iterations.
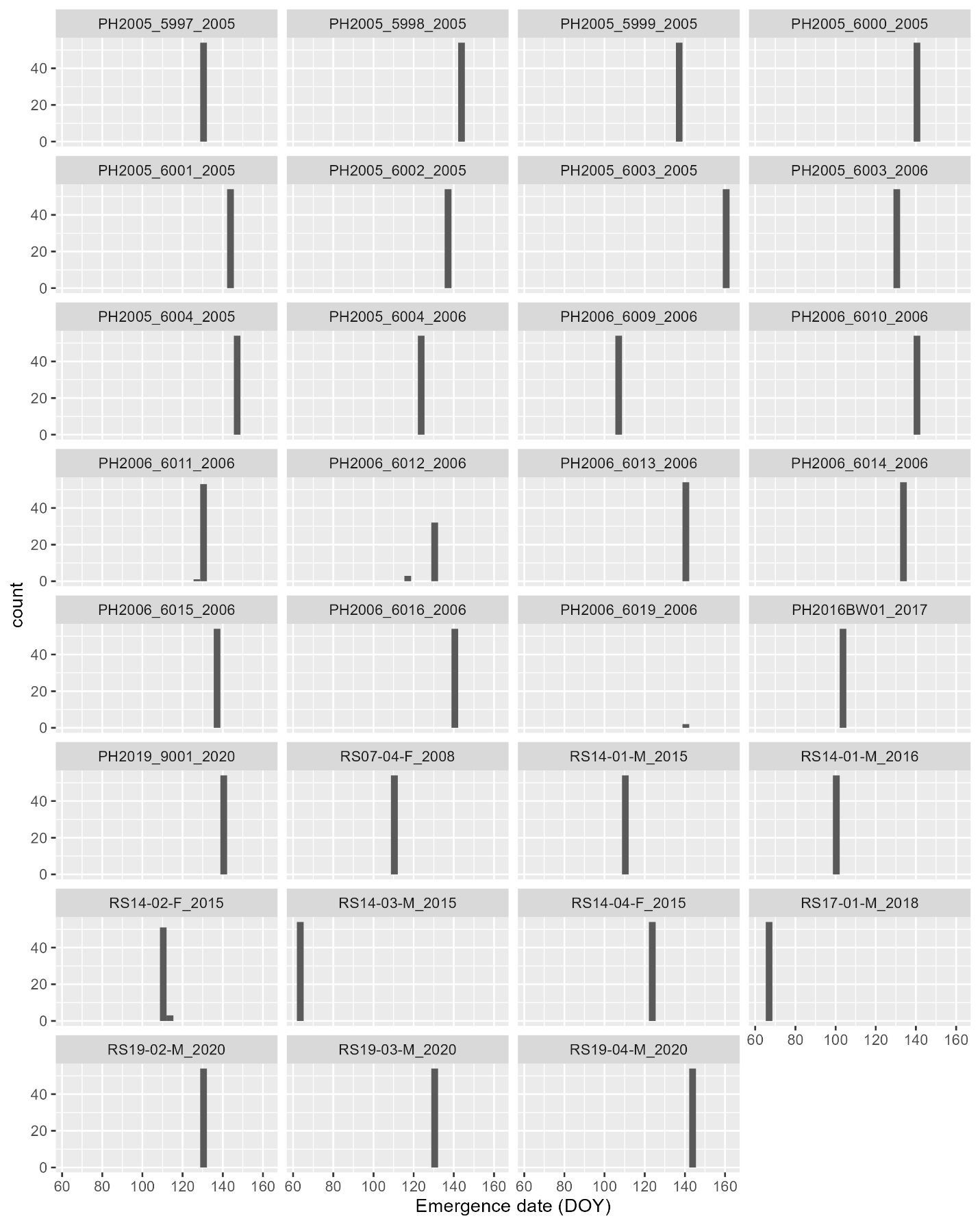


Figure S5. Viterbi-estimated emergence dates for individual subadults over all leave-one-out iterations, where the best HMM for subadult seals was re-fitted with one seal sequentially removed from the dataset. One subadult (RS08-08-M_2009) had a Viterbi-estimated emergence date when the HMM was fitted with the full dataset of subadult seals (i.e., the model presented in the main text) but did not have an estimated emergence date in some leave-one-out iterations.


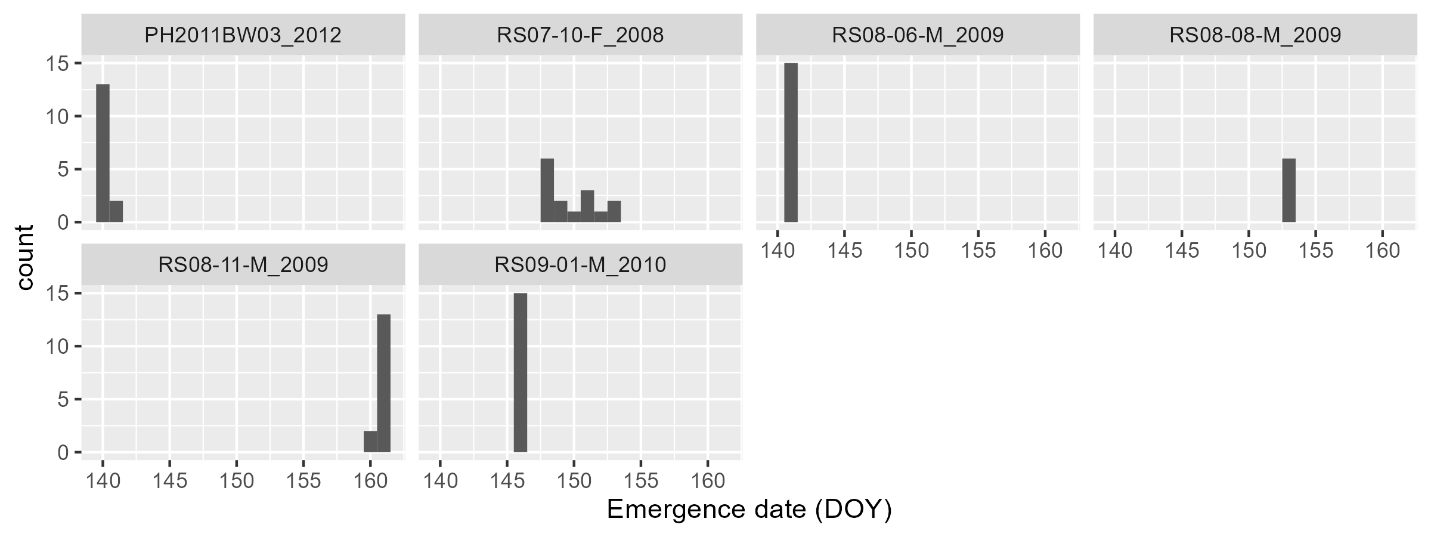

Supplement: Supplementary file 1 — Data S1: ece372948‐sup‐0001‐supinfo.docx. [file ECE3-16-e72948-s001.docx]
